# Axon guidance receptor ROBO3 modulates subtype identity and prognosis via AXL-associated inflammatory network in pancreatic cancer

**DOI:** 10.1172/jci.insight.154475

**Published:** 2022-08-22

**Authors:** Niklas Krebs, Lukas Klein, Florian Wegwitz, Elisa Espinet, Hans Carlo Maurer, Mengyu Tu, Frederike Penz, Stefan Küffer, Xingbo Xu, Hanibal Bohnenberger, Silke Cameron, Marius Brunner, Albrecht Neesse, Uday Kishore, Elisabeth Hessmann, Andreas Trumpp, Philipp Ströbel, Rolf A. Brekken, Volker Ellenrieder, Shiv K. Singh

**Affiliations:** 1 Department of Gastroenterology, Gastrointestinal Oncology and Endocrinology and; 2 Department of Gynecology and Obstetrics, University Medical Center Göttingen, Göttingen, Germany.; 3 Division of Stem Cells and Cancer, DKFZ, Heidelberg, Germany.; 4 HI-STEM: The Heidelberg Institute for Stem Cell Technology and Experimental Medicine gGmbH, Heidelberg, Germany.; 5 Department of Pathology and Experimental Therapy, School of Medicine, University of Barcelona, L’Hospitalet de Llobregat, Barcelona, Spain.; 6 Molecular Mechanisms and Experimental Therapy in Oncology Program (Oncobell), Bellvitge Biomedical Research Institute, L’Hospitalet de Llobregat, Barcelona, Spain.; 7 Department of Internal Medicine II, Klinikum rechts der Isar, Technische Universität München, Munich, Germany.; 8 Institute of Pathology,; 9 Department of Cardiology and Pneumology, and; 10 Clinical Research Unit 5002, KFO5002, University Medical Center Göttingen, Göttingen, Germany.; 11 Biosciences, College of Health, Medicine and Life Sciences, Brunel University London, Uxbridge, United Kingdom.; 12 Department of Veterinary Medicine, United Arab Emirates University, Al Ain, United Arab Emirates.; 13 Hamon Center for Therapeutic Oncology Research, Departments of Surgery and Pharmacology, UT Southwestern Medical Center, Dallas, Texas, USA.

**Keywords:** Gastroenterology, Therapeutics, Cancer, Cytokines, Molecular biology

## Abstract

Metastatic pancreatic cancer (PDAC) has a poor clinical outcome with a 5-year survival rate below 3%. Recent transcriptome profiling of PDAC biopsies has identified 2 clinically distinct subtypes — the “basal-like” (BL) subtype with poor prognosis and therapy resistance compared with the less aggressive and drug-susceptible “classical” (CLA) subtype. However, the mechanistic events and environmental factors that promote the BL subtype identity are not very clear. Using preclinical models, patient-derived xenografts, and FACS-sorted PDAC patient biopsies, we report here that the axon guidance receptor, roundabout guidance receptor 3 (ROBO3), promotes the BL metastatic program via a potentially unique AXL/IL-6/phosphorylated STAT3 (p-STAT3) regulatory axis. RNA-Seq identified a ROBO3-mediated BL-specific gene program, while tyrosine kinase profiling revealed AXL as the key mediator of the p-STAT3 activation. CRISPR/dCas9-based ROBO3 silencing disrupted the AXL/p-STAT3 signaling axis, thereby halting metastasis and enhancing therapy sensitivity. Transcriptome analysis of resected patient tumors revealed that AXL^hi^ neoplastic cells associated with the inflammatory stromal program. Combining AXL inhibitor and chemotherapy substantially restored a CLA phenotypic state and reduced disease aggressiveness. Thus, we conclude that a ROBO3-driven hierarchical network determines the inflammatory and prometastatic programs in a specific PDAC subtype.

## Graphical abstract

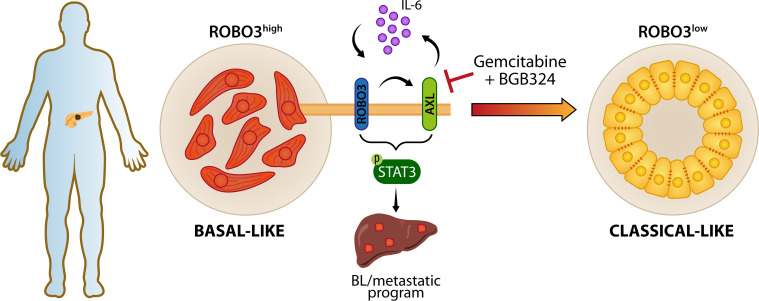

## Introduction

Pancreatic ductal adenocarcinoma (PDAC) is one of the most aggressive cancers, with a 5-year survival rate of less than 9% (1). The majority of patients with PDAC exhibit metastatic disease at the time of diagnosis, when surgical resection is no longer possible (2). For locally advanced and metastasized tumors, chemotherapy with either FOLFIRINOX or gemcitabine plus nab-paclitaxel is the preferred treatment (3–5). The complex tumor heterogeneity profoundly contributes to its grave prognosis and renders clinical management challenging (6–8). Recent whole-genome sequencing identified distinct genome-based subtypes, which are linked to mutational landscapes within DNA damage repair pathways, RNA processing, and axon guidance pathways in PDAC progression (9–11). Treatment regimens aiming at specific molecular vulnerabilities have not yet been established, except for patients with germline BRCA mutations (12, 13). Besides genomic subtypes, the identification of transcriptome-based molecular subtypes has considerably helped improve the early prognosis and therapeutic interventions in patients with PDAC (14–16). Transcriptomic profiling in PDAC tumors has revealed 2 distinct subtypes: classical (CLA) and basal-like (BL). The CLA subtype usually presents with a less aggressive clinical course and a better response to chemotherapy; BL tumors are decisively linked to poor prognosis with a pronounced resistance to chemotherapy (10, 14–18). The CLA subtype is characterized by high expression of epithelial lineage markers (i.e., GATA6), whereas the BL subtype exhibits high expression of TP63 (14, 19–21).

Recently, genome-wide molecular analyses have revealed an altered axon guidance SLIT/roundabout guidance receptor (ROBO) signaling pathway in PDAC (11, 16, 22). Although some members of the axon guidance pathway, e.g., ROBO1, ROBO2, and SLIT2, are linked to better clinical outcome in patients with PDAC (23–25), high expression of ROBO3 has been found in advanced tumors (26), correlating with poor prognosis (22). However, the functional significance of ROBO3 in PDAC plasticity and tumor progression are poorly understood.

In the present study, we examined whether activation of the axon guidance pathway plays a role in PDAC subtype specification and whether this offers an option for future treatment strategies. Here, we show that ROBO3 promotes the formation of a highly metastatic and chemoresistant BL subtype. Mechanistically, ROBO3 induces transcription of BL-associated gene signatures — at least in part — through what we believe is a previously undescribed AXL/phosphorylated STAT3 (p-STAT3) pathway. Genetic or pharmacological inactivation of ROBO3/AXL/p-STAT3 signaling through CRISPR/dCas9 technology or administration of BGB324, a clinical stage AXL inhibitor, reduced ascites as well as liver metastases in vivo and restored chemosensitivity in vitro in BL subtype-specific PDAC. Together, our study uncovers a mechanism in the regulation of PDAC subtype specification. In addition, it supports current therapeutic concepts in pancreatic cancer aiming at subtype interference to overcome therapy resistance.

## Results

### Axon guidance receptor ROBO3 is linked to the BL PDAC subtype.

**Figure 1:**
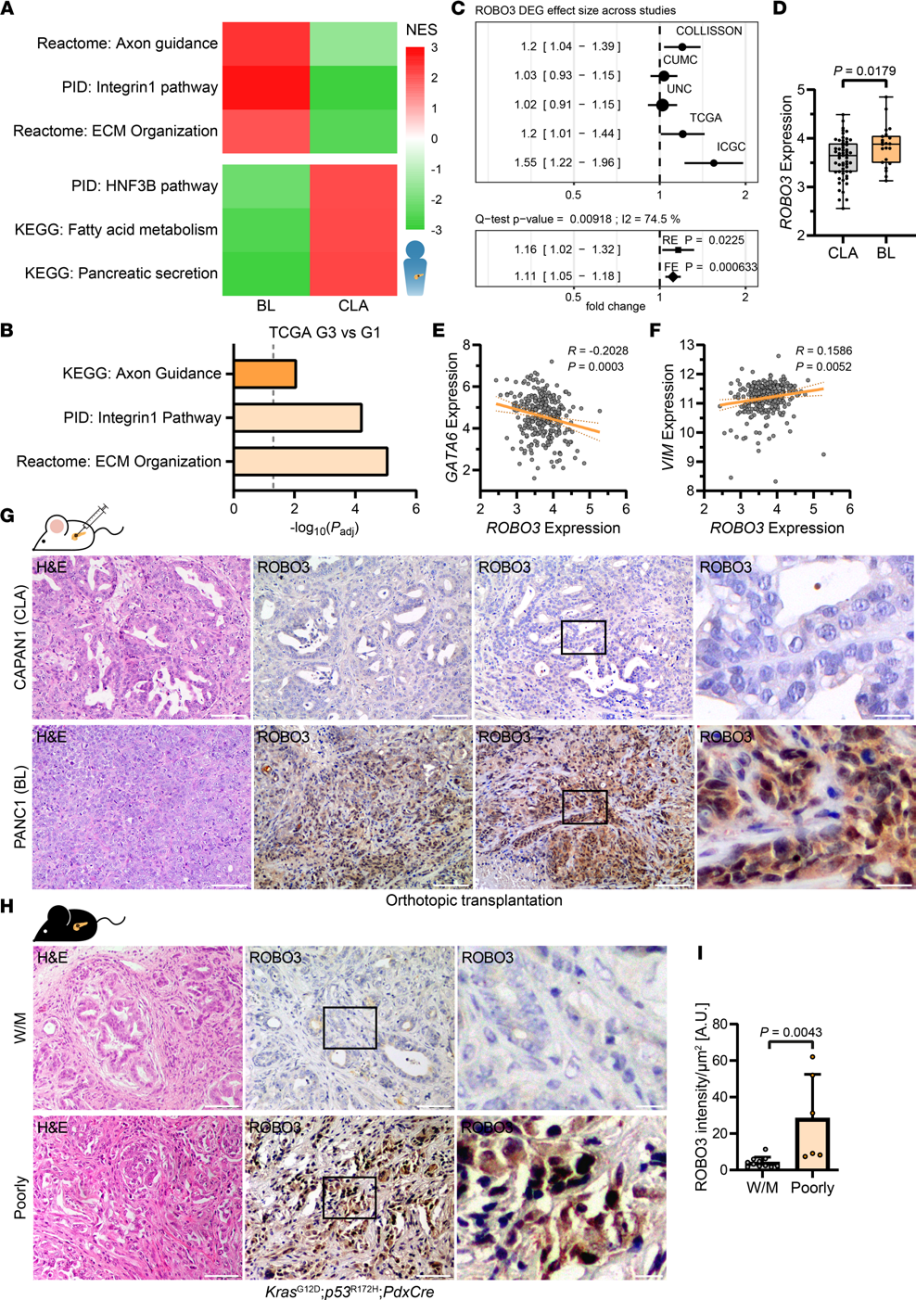
High ROBO3 expression correlates with BL/poorly differentiated phenotype. (**A**) Heatmap of normalized enrichment scores (NESs) of selected pathways in basal-like (BL) and classical (CLA) PDAC patient microarray data (17). (**B**) Gene set overrepresentation analysis of TCGA patient data (10) between high-grade G3 (*n* = 48) and low-grade G1 tumors (*n* = 31). Data were retrieved and differential analysis was performed using R2 platform. (**C**) Meta-analysis of ROBO3 across PDAC patient cohorts (10, 15, 18, 28, 29). See Methods for definitions. The effect size is determined in differential gene expression analyses between squamous/QM/BL and CLA/progenitor PDAC. (**D**) rma function–normalized expression of ROBO3 in CLA (*n* = 56) and BL (*n* = 22) PDAC patient microarray data with high tumor cellularity (17). Box (25th to 75th percentile with median) and whiskers (min to max) are shown. (**E** and **F**) Correlation of ROBO3 and GATA6 (**E**), and ROBO3 and VIM (**F**), expression in PDAC patient microarray data (17). rma-normalized probe intensities and linear regression with 95% CI are shown. *n* = 309. (**A** and **D**–**F**) Data were accessed from ArrayExpress (E-MTAB-6134). (**G**) Representative H&E (left) and IHC staining for ROBO3 in orthotopically implanted CLA (CAPAN1) and BL (PANC1) cell lines in the pancreas of NMRI-*Foxn1^nu/nu^* mice. Right panel shows higher magnification of indicated area. Scale bar: 50 μm, magnified area (right), 10 μm. (**H**) Representative H&E (left) and IHC staining of ROBO3 (middle and right) in *Kras*^G12D^ *p53*^R172H^ *PdxCre* (KPC) tumors. Right: higher magnification of indicated area. Scale bar 50 μm, magnified area (right), 10 μm. (**I**) ROBO3 staining intensity of **H**. Scatterplots show average intensity per field of view (F.o.V.) per mouse, as arbitrary units (AU) with means ± SD. Mann-Whitney test. W/M (G1 and G2), *n* = 11; poorly (G3 and G4), *n* = 6. DEG, differentially expressed gene; RE, random effect; FE, fixed effect.

In view of recent studies suggesting a role of axon guidance signaling in PDAC progression (10, 22, 27), we interrogated how activation of the axon guidance pathway could affect disease aggressiveness, prognosis, and subtype specificity. We initially used publicly available data sets of PDAC patient biopsies (15, 17). A significant enrichment of axon guidance–associated gene sets with acquisition of a BL subtype was noted, where integrin1 pathway genes and ECM organization gene signatures were also induced (Figure 1A), as in poorly differentiated high-grade G3 tumors (ref. 15; Figure 1B). These findings prompted us to study axon guidance receptor signaling in PDAC subtype specification. We focused on the ROBO3 receptor protein that has previously been linked to poor prognosis in PDAC (22, 26). In fact, effect size meta-analysis across multiple human PDAC data sets (10, 15, 28, 29) supported differential gene expression of ROBO3 in squamous/QM/BL (BL) and CLA/progenitor (CLA) PDAC subtypes. Our analyses revealed that ROBO3 expression was markedly higher in BL PDAC tumors (Figure 1C). We then examined ROBO3 expression in the CLA and BL subtypes in human PDAC specimens, where BL tumors were strongly associated with a poorly differentiated and metastatic phenotype (17, 30); we found significantly higher expression of ROBO3, particularly in BL PDAC tumors (Figure 1D). Within this data set, we observed that ROBO3 expression correlated negatively with the CLA marker, GATA6 (Figure 1E), while BL/epithelial-mesenchymal transition (EMT) VIM expression positively correlated in PDAC patient tumors (Figure 1F). Therefore, we experimentally validated the subtype-specific expression of ROBO3 in PDAC cell lines as well as in vivo following orthotopic implantation of these cells. We used CLA CAPAN1 and CAPAN2 cell lines, which express CLA/epithelial lineage genes, as well as PANC1 and MiaPaCa2, which show BL/EMT lineage gene signatures (29, 31, 32). We found a strong ROBO3 expression in these BL cell lines compared with the CLA ones (Supplemental Figure 1A; supplemental material available online with this article; https://doi.org/10.1172/jci.insight.154475DS1). Next, we used an orthotopic mouse model derived from these CLA (i.e., CAPAN1, ROBO3^lo^) and BL (i.e., PANC1, ROBO3^hi^) PDAC cell lines (Figure 1G). The orthotopic tumors derived from CAPAN1 recapitulated well to moderately (W/M) differentiated CLA tumors (Figure 1G, left upper) with high GATA6 and low VIM expression (Supplemental Figure 1B). PANC1 tumors exhibited a poorly differentiated/BL phenotype (Figure 1G, left lower) with low GATA6 and high VIM expressions (Supplemental Figure 1B). As expected, we found that ROBO3 expression was elevated in BL/poorly differentiated orthotopic tumors compared with the CLA subtype (Figure 1G), consistent with the high ROBO3 expression in BL/poorly differentiated patient tumors (Figure 1, C and D). To confirm this association, we used the well-established *Kras^G12D^* *p53^R172H^* *PdxCre* (KPC) mouse model, which recapitulates the entire spectrum of human PDAC tumors (33, 34), ranging from well to poorly differentiated phenotypic states. KPC-derived PDAC tumors were histologically categorized into W/M differentiated/CLA (G1 and G2) and poorly differentiated/BL (G3 and G4) groups (Figure 1H), as described previously (30). Indeed, KPC-derived W/M tumors expressed high GATA6 and low VIM, whereas poorly differentiated tumors exhibited low GATA6 and high VIM levels (Supplemental Figure 1C). We then probed ROBO3 in these KPC tumors; consistent with our findings in human PDAC tissues and cell lines as well as orthotopic PDAC models, we found a strong positive correlation between high ROBO3 expression levels and acquisition of a poorly differentiated BL PDAC subtype (Figure 1, H and I).

### ROBO3 promotes lineage-specific program to maintain BL aggressive subtype.

**Figure 2:**
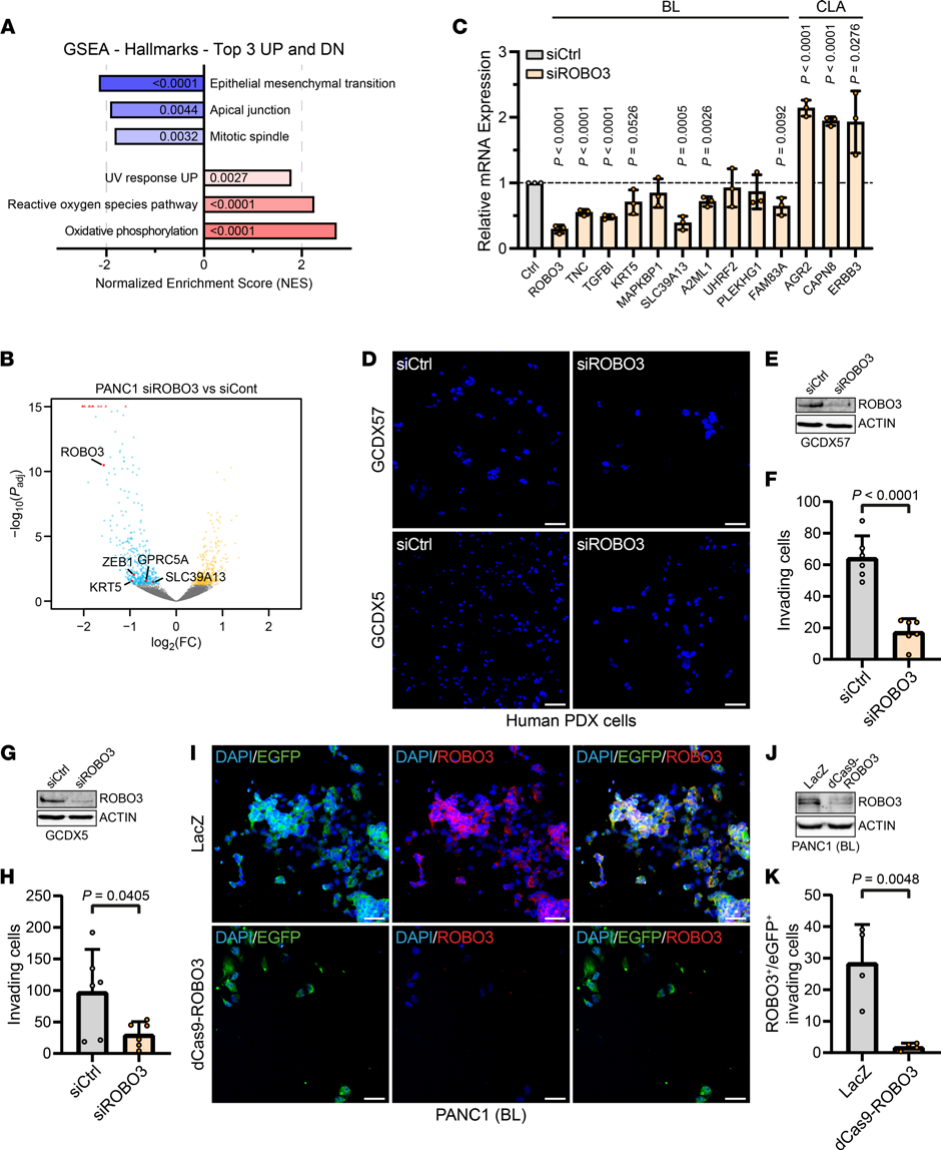
BL transcriptome signatures are associated with ROBO3 expression. (**A** and **B**) RNA-Seq was performed on BL PANC1 cells transfected with ROBO3 targeting (siROBO3) or control siRNA (siCtrl); *n* = 3. (**A**) Gene set enrichment analysis (GSEA) of the top 3 altered hallmark gene signatures of the Molecular Signatures Database (MSigDB) collection after ROBO3 knockdown. The bar graphs show normalized enrichment scores (NESs). Significance is indicated by the FDR *q* values. (**B**) Volcano plot of differential expression analysis. Downregulated genes coinciding with a known BL signature are plotted in red. (**C**) Quantitative real-time PCR (qRT-PCR) analysis of selected BL and CLA genes, based on the published data of Collisson et al. (29), Moffitt et al. (18), and Bailey et al. (10), in PANC1 cells. Results show average relative quantification (to control treatment) ± SD. Significance was determined by an unpaired Student’s *t* test. *n* = 3. (**D**–**H**) Transwell invasion assay of ROBO3^hi^-classified GCDX57 and GCDX5 PDX-derived cell lines transfected with siROBO3 or siCtrl. (**D**) Representative DAPI staining of invaded cells. Scale bar: 50 μm. (**E** and **G**) Immunoblot for ROBO3 and β-actin as loading control for GCDX57 (**E**) and GCDX5 (**G**). (**F** and **H**) Quantification of **D** for GCDX57 (**F**) and GCDX5 (**G**) cells. Scatterplots show average counts as well as means ± SD as bar graphs. Statistical significance was determined by an unpaired Student’s *t* test. *n* = 6. (**I**–**K**) Transwell invasion assay of BL PANC1 cells with CRISPR/dCas9-mediated knockdown of ROBO3 and LacZ control cells. (**I**) Representative IF staining for EGFP and ROBO3 of invaded cells. Scale bar: 50 μm. (**J**) Immunoblot for ROBO3 and β-actin as loading control in LacZ control and dCas9-ROBO3 PANC1 cells. (**K**) Quantification of ROBO3 and EGFP double-positive cells of **I**. Scatterplots show average counts as well as means ± SD as bar graphs. Statistical significance was determined by an unpaired Student’s *t* test. *n* = 4. PDX, patient-derived xenograft; IF, immunofluorescence.

We next examined whether ROBO3 signaling was involved in transcriptional determination of BL subtype specification and functions. We therefore performed RNA-Seq analysis following ROBO3 silencing in the BL cell line, PANC1 (Figure 2, A and B; and Supplemental Figure 2, A–C), and analyzed gain and loss of PDAC subtype–specific hallmarks (14, 27, 35). In support of our notion that ROBO3 controlled subtype-specific features, we found a significant loss of BL-associated hallmark gene sets, e.g., EMT, apical junction, and mitotic spindle, upon ROBO3 silencing in BL cells (Figure 2A). Moreover, ROBO3 silencing caused reduced expression of the BL marker genes KRT5, ZEB1, SCL39A13, and GPRC5A (Figure 2B), whereas CLA signatures (e.g., oxidative phosphorylation and reactive oxygen species pathways) were enriched in BL PDAC cells (Figure 2A). Results from GSEA were confirmed by qRT-PCR (Figure 2C), suggesting that ROBO3 signaling controlled PDAC subtype plasticity in favor of a BL phenotype.

Next, we examined the functional implications of ROBO3 signaling in BL subtype features, and thus, conducted 3D invasion and cell viability assays using a series of ROBO3^hi^ human PDAC cell lines upon genetic depletion of the receptor. siRNA-mediated silencing of ROBO3 did not affect tumor cell viability (data not shown), but tumor cell invasiveness was significantly reduced in PDX-derived primary PDAC cell lines (Figure 2, D–H). We then established a CRISPR/dCas9/EGFP-based method to stably silence ROBO3 in PDAC cell lines. We verified a series of stable dCas9-ROBO3 and LacZ control clones at protein and mRNA levels using the BL ROBO3^hi^ PANC1 cells (Supplemental Figure 2, D and E). Consistent with the siRNA results, stable silencing of ROBO3 in dCas9-ROBO3 BL cells significantly reduced the invasion capacity of BL (PANC1) cells (Figure 2, I–K). Interestingly, loss of ROBO3 also restored PDAC cell responsiveness to chemotherapy (Supplemental Figure 2F). Together, these experiments demonstrate that ROBO3 signaling controls PDAC subtype–specific features at the transcriptional and functional levels, thereby promoting the acquisition of an aggressive and therapy-resistant PDAC subtype.

### ROBO3 deficiency reduces metastasis and prolongs survival.

**Figure 3:**
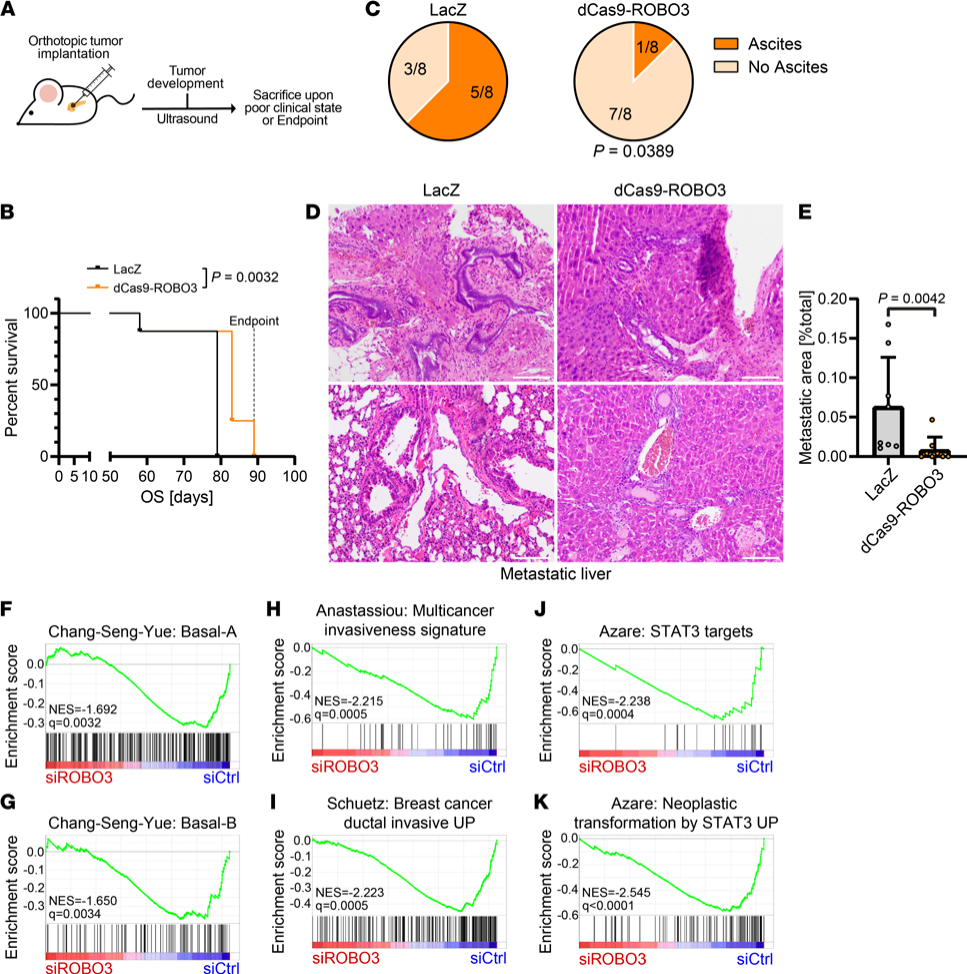
Genetic inactivation of ROBO3 is associated with reduced metastasis. (**A**) Experimental design for orthotopic implantation of LacZ control and dCas9-ROBO3 PANC1 cells in immunocompromised NMRI-*Foxn1^nu/nu^* mice. (**B**) Kaplan-Meier graph for survival analysis comparing LacZ control and dCas9-ROBO3 cohorts. Significance determined by log-rank (Mantel-Cox) test. (**C**) Ascites rates of mice bearing orthotopic LacZ control or dCas9-ROBO3 tumors. Significance was determined by χ^2^ test. (**D**) Representative H&E staining of liver metastases in mice bearing orthotopic LacZ control or dCas9-ROBO3 tumors. Scale bar: 50 μm. (**E**) Metastatic area in mice bearing orthotopic LacZ control and dCas9-ROBO3 tumors. Scatterplots show the metastatic area as percentage of the total evaluated liver area as well as means ± SD as bar graphs. Significance was determined by Mann-Whitney test. (**A**–**E**) LacZ control, *n* = 8; dCas9-ROBO3, *n* = 8. (**F**–**K**) Gene set enrichment analysis (GSEA) of ROBO3-silencing RNA-Seq data in PANC1 cells for genes corresponding to published PDAC subtypes (ref. 14; **F** and **G**), as well as selected gene sets of the curated Molecular Signatures Database (MSigDB) collection (**H**–**K**). Normalized enrichment scores (NESs) and FDR *q* values are indicated.

To evaluate whether ROBO3 signaling maintained BL tumor progression, we orthotopically implanted dCas9-ROBO3 and LacZ control BL (PANC1) cells into the pancreas of immunodeficient mice (Figure 3A). PANC1-derived orthotopic tumors are known to form highly invasive and metastatic PDAC. We observed that ROBO3 promoted tumor progression, and therefore, genetic inactivation of ROBO3 was associated with reduced ascites, reduced metastasis, and a substantial increase in survival (Figure 3, B–E). Notably, 5 our of 8 LacZ control mice and only 1 out of 8 dCas9-ROBO3 silenced mice developed malignant ascites (Figure 3C), a strong indicator of peritoneal carcinomatosis and tumor progression. Histopathological examinations of the liver tissues further revealed a significant reduction in hepatic metastatic burden following ROBO3 silencing (Figure 3, D and E).

Next, we analyzed the likely molecular signatures involved in ROBO3-driven PDAC aggressiveness and metastatic progression. Intriguingly, our curated GSEA revealed that ROBO3 silencing was associated with a significant inactivation of gene programs involved in BL-specific metastatic signatures (ref. 14; Figure 3, F and G), invasiveness (Figure 3, H and I), and inflammation such as STAT3 signaling pathway (Figure 3, J and K).

### ROBO3 maintains p-STAT3^Y705^ activity in BL/high-grade PDAC tumors.

**Figure 4:**
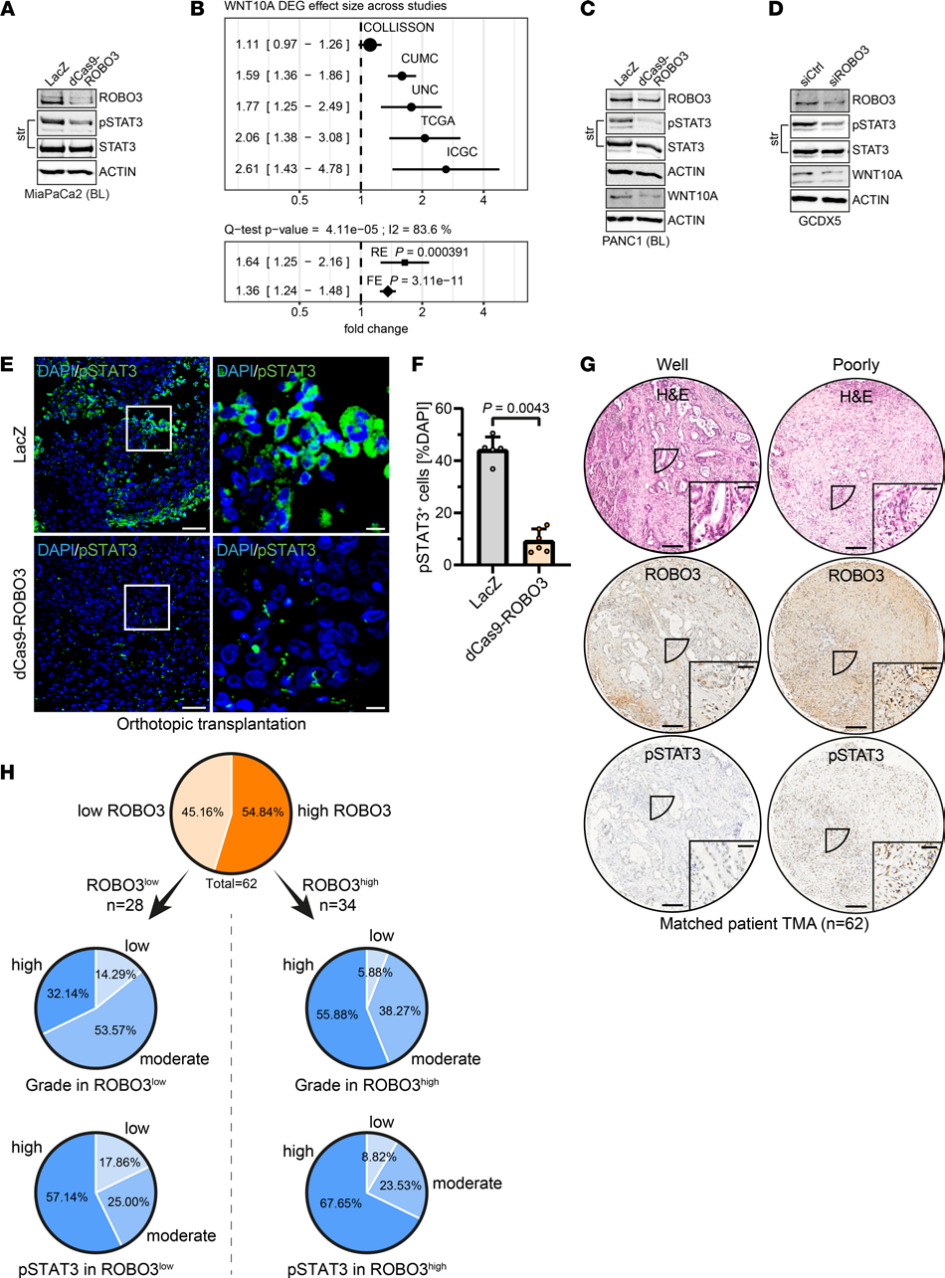
ROBO3 maintains STAT3 activity in high-grade PDAC tumors. (**A**) Immunoblot for ROBO3, Y705-phosphorylated STAT3 (p-STAT3), and total STAT3, as well as β-actin as loading control, in LacZ control and dCas9-ROBO3 MiaPaCa2 cells. Representative of *n* = 3 independent experiments. (**B**) Differentially expressed gene (DEG) effect size of WNT10A across the indicated PDAC patient cohorts (10, 15, 27, 28). (**C** and **D**) Immunoblot for ROBO3, p-STAT3, STAT3, and WNT10A, as well as β-actin as loading control, in LacZ control and dCas9-ROBO3 PANC1 cells (**C**) as well as in ROBO3^hi^-classified GCDX5 cells (**D**) following siRNA-mediated knockdown of ROBO3 (siROBO3) or control siRNA (siCtrl). Representative of *n* = 3 independent experiments. (**E**) Representative immunofluorescence (IF) staining for p-STAT3 in primary LacZ and dCas9-ROBO3 orthotopic tumors. Scale bar: 50 μm. (**F**) Quantification of **E**. Scatterplots show the number of p-STAT3^+^ cells as percentage of DAPI^+^ cells per animal as well as means ± SD as bar graphs. Statistical significance was determined by Mann-Whitney test. LacZ, *n* = 5; dCas9-ROBO3, *n* = 6. (**G**) Representative H&E and IHC staining for ROBO3 and p-STAT3 of tumor microarray (TMA) spots of primary PDAC tissue derived from 62 human PDAC patients’ resected tissue, showing matched tumor tissues in each column. Scale bar: 200 μm, for magnified area, 50 μm. (**H**) Evaluation of ROBO3 intensity (scale 0–3) and p-STAT3 IHC immunoreactive scores (IRSs, scale 0–12) as well as grading of TMA spots. Top, all 62 patients were separated into low ROBO3 (intensity < 2, *n* = 28) and high ROBO3 (intensity ≥ 2, *n* = 34). Middle, grading in ROBO3^lo^ (left) and ROBO3^hi^ (right) patients. Low, G1–2; moderate, G2; high, G2–3/3. Bottom, p-STAT3 level in ROBO3^lo^ (left) and ROBO3^hi^ (right) patients. Low, IRS < 6; moderate, 6 ≤ IRS < 8; high, IRS ≥ 8. Histopathological grading performed by expert pathologists. A total of 1–3 TMA spots were evaluated and averaged per patient for intensity and IRSs. *n* = 62.

We next examined whether ROBO3-mediated BL subtype specificity was mechanistically linked to the STAT3 signaling pathway. Of note, IL-6/STAT3 activation significantly contributes to metastatic spread in PDAC (36). We thus measured the phosphorylation/activation status of STAT3 at Y705 and S727 in the presence or absence of ROBO3. p-STAT3^Y705^ activity was markedly reduced in dCas9-ROBO3 BL cells (Figure 4A), whereas p-STAT3^S727^ status or total STAT3 levels were unchanged (Figure 4A and Supplemental Figure 3A). To further validate ROBO3-mediated p-STAT3^Y705^ activation for BL subtype plasticity, we transiently overexpressed ROBO3 in CLA PDAC cell lines. ROBO3 overexpression led to a significant induction of p-STAT3^Y705^ and BL subtype-specific gene signatures (e.g., SCL39A13) in CLA cell lines (Supplemental Figure 3, B–E, and Supplemental Table 3). We therefore focused on Y705 phosphorylation (hereafter referred to as p-STAT3) and its role in ROBO3-mediated tumor progression. Moreover, the relevance of ROBO3-dependent STAT3 activation in BL subtype identity and plasticity was supported by decreased expression of the p-STAT3 downstream target WNT10A, a member of the canonical WNT pathway and driver of EMT-related tumor cell invasion (35). We noted that WNT10A expression positively correlated with the BL subtype identity in the aforementioned PDAC databases (10, 15, 28, 29; Figure 4B). We observed a robust expression of p-STAT3 and WNT10A in the BL PDAC cell lines, in which inactivation of ROBO3 caused reduced p-STAT3 and WNT10A expression levels (Figure 4, C and D). Moreover, orthotopic implantation of ROBO3^hi^ BL subtype cells (LacZ cells) formed metastatic PDAC with high p-STAT3 levels (Figure 4, E and F). Consistent with our in vitro data, genetic inactivation of ROBO3 not only caused loss of BL-specific gene signatures (Figure 3, F–I) but also resulted in reduced STAT3 gene signatures (Figure 3, J and K) and its phosphorylation (Figure 4A). Finally, we stained a series of ROBO3^hi^ and ROBO3^lo^ human PDAC tumors (*n* = 62) for p-STAT3 and STAT3 expression (Figure 4, G and H; and Supplemental Figure 3, F and G). Importantly, these data supported high expression of p-STAT3 and ROBO3 particularly in high-grade/poorly differentiated PDAC tumors (Figure 4, G and H), while total STAT3 expression was relatively unchanged (Supplemental Figure 3, F and G).

### IL-6–dependent STAT3 phosphorylation is disrupted in ROBO3-deficient BL cells.

**Figure 5:**
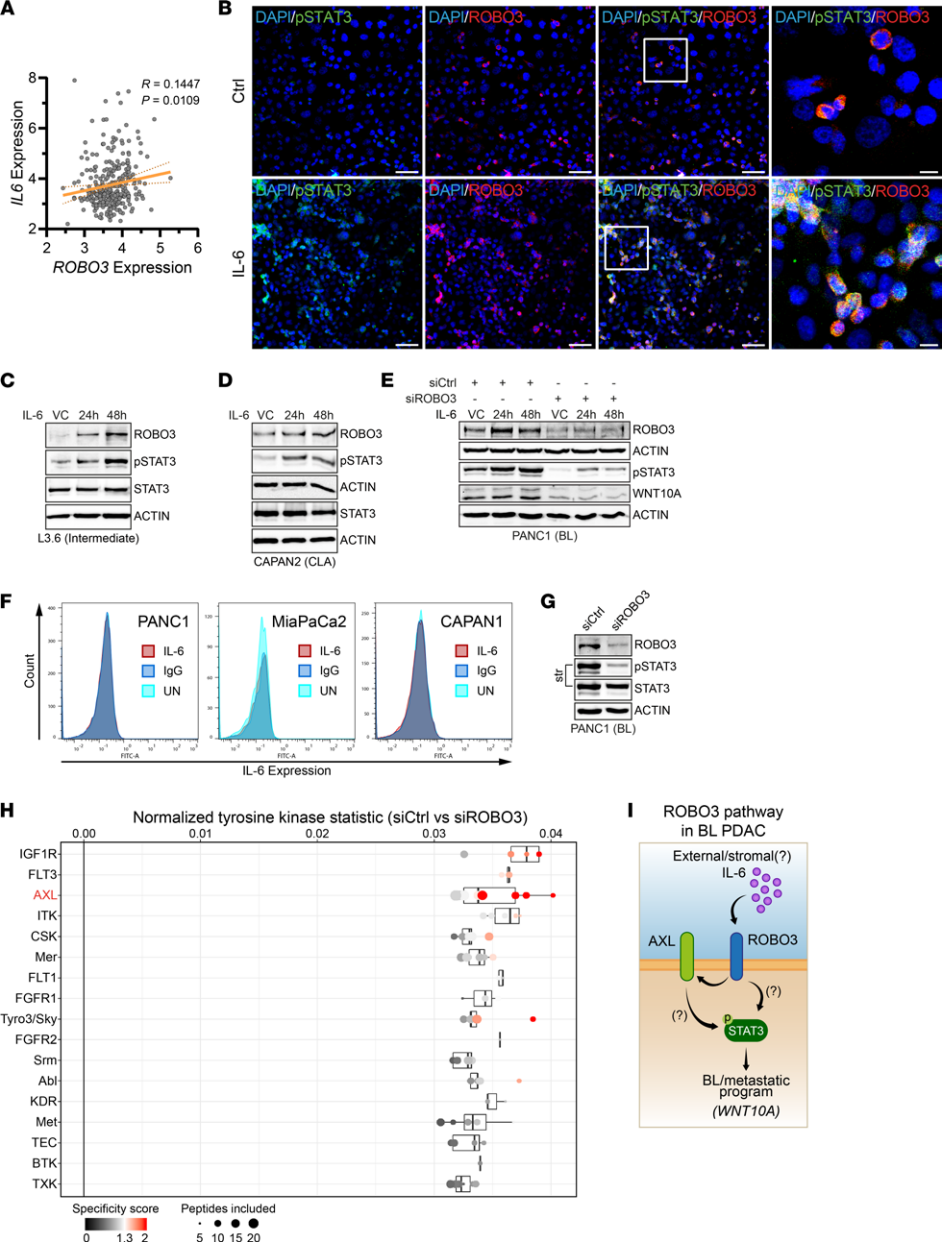
IL-6–independent activation of STAT3 in BL PDAC cells. (**A**) Correlation of ROBO3 and IL-6 expression in PDAC patient microarray data (17). rma-normalized probe intensities and linear regression with 95% CI are shown. Data was accessed from ArrayExpress (E-MTAB-6134). *n* = 309. (**B**) Representative immunofluorescence (IF) staining for Y705-phosphorylated STAT3 (p-STAT3) and ROBO3 following IL-6 treatment for 48 hours or vehicle control (VC) in BL PANC1 cells. Scale bar: 50 μm, magnified area (right panel), 10 μm. *n* = 6. (**C** and **D**) Immunoblot for ROBO3, p-STAT3, STAT3, and β-actin as loading control, in intermediate (L3.6; **C**) and CLA (CAPAN2; **D**) cell lines following IL-6 treatment for 24 and 48 hours or VC. (**E**) Immunoblot for ROBO3, p-STAT3, STAT3, WNT10A, and β-actin as loading control, in BL PANC1 cells transfected with ROBO3-targeting (siROBO3) or control siRNA (siCtrl), additionally treated with IL-6 for 24 and 48 hours or VC. (**F**) Flow cytometry of PANC1, MiaPaCa2, and CAPAN1 cells for IL-6 expression using an anti–IL-6 antibody, with isotype control and without any staining (UN). Count of gated cells are shown against fluorescence intensity, which reflects IL-6 expression. *n* = 3. (**G**) Immunoblot for ROBO3, p-STAT3, STAT3, and β-actin as loading control, in BL PANC1 cells transfected with siROBO3 or siCtrl. Equal lysates used for **H**. (**C**–**E** and **G**) Representative of *n* = 3 independent experiments. (**H**) Tyrosine (Tyr) kinase activity assay in BL PANC1 cells transfected with siROBO3 or siCtrl. Plot shows putative upstream Tyr kinases ranked by their final score (*q*). The top ranked Tyr kinases, including AXL, are more active in siCtrl compared with siROBO3. Points represent the individual analysis with a varying rank cutoff for adding upstream kinases for peptides. The size of the peptide set used for the corresponding analysis is depicted by the size of the dot. The specificity score is indicated by the red color. (**I**) Model of ROBO3-dependent induction of BL/metastatic gene expression via phosphorylation of STAT3.

To identify external factors involved in ROBO3 activation in the BL subtype, we first speculated that IL-6, which is a strong inducer of STAT3 activity, likely governed ROBO3 expression. Thus, using publicly available data on PDAC patient tumors (17), we found a positive correlation between IL-6 and ROBO3 expression (Figure 5A). To examine an involvement of IL-6 in ROBO3-mediated STAT3 activation, we treated BL subtype cells exogenously with IL-6, which induced coexpression of p-STAT3 and ROBO3 in immunocytochemistry staining, as well as nuclear localization of p-STAT3 (Figure 5B and Supplemental Figure 4A). We validated this finding in ROBO3^lo^-expressing CLA cells, where we found a significant induction of ROBO3 concomitantly with an increase in p-STAT3 following IL-6 treatment (Supplemental Figure 4, B and C). Next, we treated multiple PDAC cell lines with exogenous IL-6, which induced ROBO3 as well as p-STAT3 in a time-dependent manner (Figure 5, C and D, and Supplemental Figure 4D). These data suggest a feed-forward mechanism driven by IL-6 that activates the ROBO3/p-STAT3 axis in PDAC cells. We also examined the effect of IL-6 on this feed-forward loop with/without ROBO3 in BL PANC1 and MiaPaCa2 cells. Upon transient silencing of ROBO3, IL-6 weakly induced p-STAT3 and WNT10A expression (Figure 5E and Supplemental Figure 4E). To validate whether endogenous IL-6 interfered with ROBO3, we first examined IL-6 protein expression in the PDAC cell lines; however, none of the PDAC cell lines expressed IL-6 (Figure 5F). Although exogenous IL-6 can certainly be an inducer of ROBO3, it is not an autonomous mechanism in the BL neoplastic cells. Thus, we considered if ROBO3 may phosphorylate STAT3 via other receptor tyrosine kinase (RTK) signaling pathways in PDAC cells. We performed multiplex profiling of protein tyrosine kinase substrates in 3 independent controls as well as ROBO3-silenced BL PDAC cells (Figure 5, G and H, and Supplemental Figure 5A). The top significantly reduced kinase activity of RTKs following ROBO3 silencing in BL PDAC cells was observed for IGF1R, FLT3, and AXL (Figure 5H). Among these, we focused on AXL because it is associated with liver metastases and chemoresistance in PDAC (37, 38). To assess whether ROBO3 maintained p-STAT3 activity via AXL, we silenced ROBO3 in BL cell lines, which caused a marked reduction in AXL, p-STAT3, and WNT10A in both BL cell lines following ROBO3 silencing (Supplemental Figure 5, B and C). Thus, ROBO3 positively regulates the AXL RTK to stabilize p-STAT3 in an IL-6–independent fashion in PDAC cells (Figure 5I).

### ROBO3-mediated AXL expression associates with p-STAT3 activity in BL cells.

**Figure 6:**
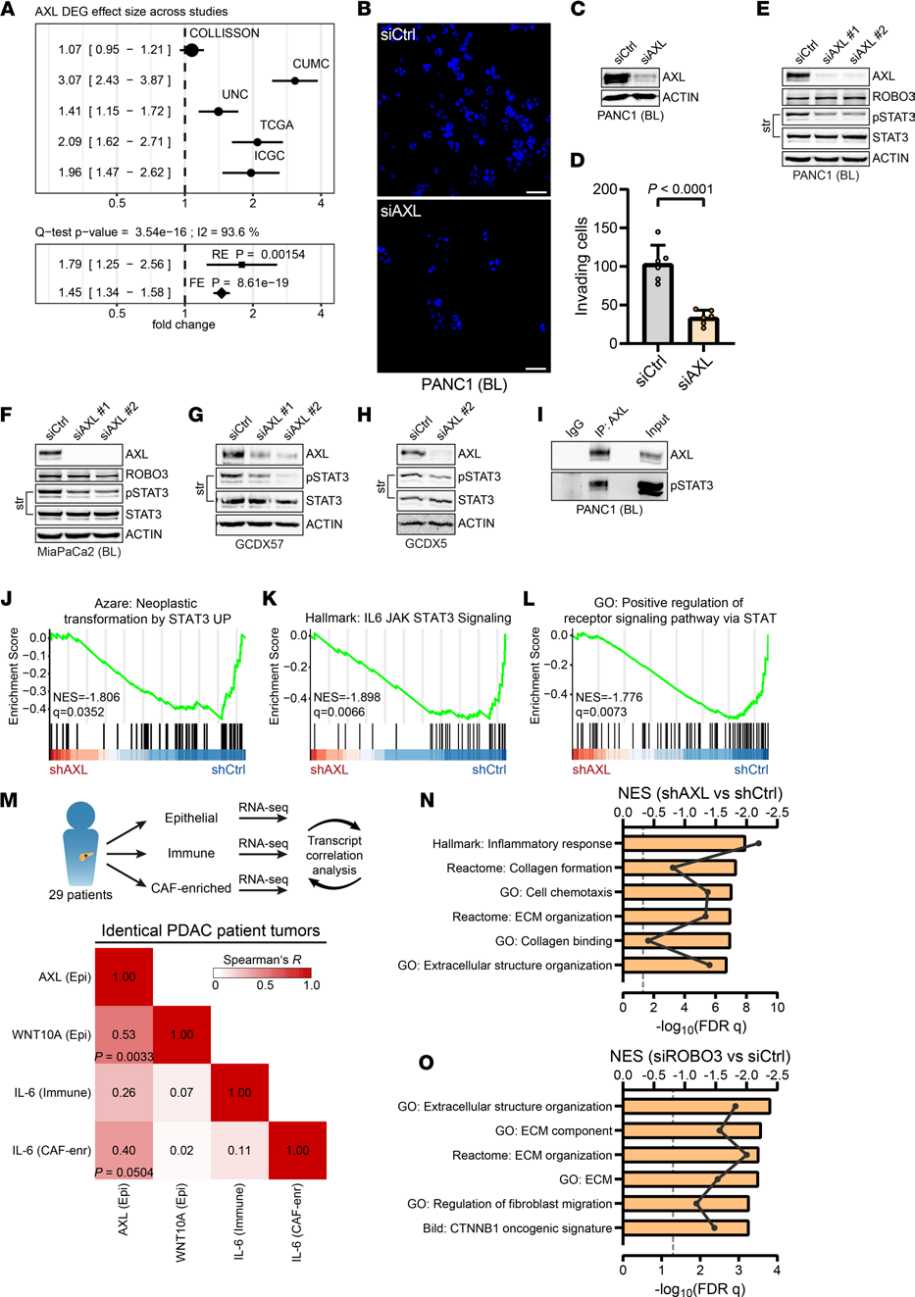
ROBO3 exploits AXL tyrosine kinase for STAT3 phosphorylation in BL cells. (**A**) DEG effect size of AXL across PDAC patient cohorts (10, 15, 18, 28, 29). (**B**–**D**) Invasion assay of BL PANC1 cells transfected with AXL (siAXL) or control siRNA (siCtrl). (**B**) Representative DAPI staining of invaded cells. Scale bar: 50 μm. (**C**) Immunoblot for AXL and β-actin. (**D**) Quantification of **B**. Scatterplots show average counts and means ± SD. Statistical significance was determined by an unpaired Student’s *t* test. *n* = 6. (**E** and **F**) Immunoblot for AXL, ROBO3, p-STAT3, total STAT3, and β-actin, in BL PANC1 (**E**) and MiaPaCa2 (**F**) cells transfected with siAXL or siCtrl. (**G** and **H**) Immunoblot for AXL, p-STAT3, STAT3, and β-actin, in ROBO3^hi^-classified GCDX57 (**G**) and GCDX5 (**H**) cells transfected with siAXL or siCtrl. (**I**) Immunoblot of co-immunoprecipitation of AXL for AXL and p-STAT3 in AXL pulldown, IgG control, and input. (**C** and **E**–**I**) Representative of *n* = 3 independent experiments; β-actin as loading control. (**J**–**L**) GSEA of RNA-Seq data for shRNA depletion of AXL (shAXL) and control shRNA (shCtrl) (ref. 38; GEO GSE128417) for selected MSigDB gene sets. NESs and FDR *q* values are indicated. (**M**) Transcript correlation analysis of compartment-specific transcriptional profiles of PDAC patients. Tumor resection material was FACS-sorted into epithelial (EPCAM^+^CD45^–^; *n* = 29; “Epi”), immune (EPCAM^–^CD45^+^; *n* = 27), and CAF-enriched (EPCAM^–^CD45^–^; *n* = 7; “CAF-enr”) and RNA-Seq performed. Spearman’s correlation (indicated by color) was calculated from epithelial AXL to epithelial WNT10A and immune and CAF-enriched IL-6. Significant correlations are indicated by their *P* values. (**N** and **O**) GSEA of RNA-Seq data for shAXL versus shCtrl (**N**; as in **J**–**L**) and for siROBO3 versus siCtrl (**O**; as in Figure 3, F–K) for stroma-related MSigDB gene sets. NESs are shown in bar graphs, and –log10 FDR *q* values are indicated as line graphs. str, stripped; GO, Gene Ontology.

AXL-mediated oncogenic functions have been linked to disease aggressiveness and therapy resistance in breast cancer and PDAC (37–40). However, AXL-mediated tyrosine kinase activation of p-STAT3 activity in the context of ROBO3 has not been previously reported in PDAC or other tumors. Here, AXL expression positively associated with the BL PDAC tumor subtype (Figure 6A). In addition, AXL depletion recapitulated the ROBO3-deficient phenotype in PDAC cells. AXL depletion rendered BL cells less invasive (Figure 6, B–D; and Supplemental Figure 6, A–C), while partially restoring chemosensitivity to gemcitabine (Supplemental Figure 6, D and E). We next examined whether AXL directly regulated p-STAT3 activity; therefore, we transiently silenced AXL in both established BL and PDX cell lines. As expected, we found a marked reduction in p-STAT3 levels, whereas total STAT3 level remained unchanged following AXL silencing (Figure 6, E–H). These results were consistent with the ROBO3-deficient phenotypic characteristics observed in BL PDAC cells (Figure 2, D–K). Interestingly, co-immunoprecipitation experiments demonstrated that AXL and p-STAT3 show protein-protein interaction in ROBO3^hi^ BL cells (Figure 6I), supporting the idea of a ROBO3-driven AXL/p-STAT3 signaling axis in BL PDAC. We reanalyzed STAT3 target gene signatures upon shRNA-mediated AXL silencing in a publicly available RNA-Seq data set (41). As anticipated, multiple STAT3 target gene sets were markedly reduced upon AXL silencing, including IL-6/JAK/STAT3 signaling (Figure 6, J–L). Thus, the ROBO3/AXL regulatory axis can maintain STAT3 activation in neoplastic epithelial cells in the absence of exogenous IL-6. In the tumor microenvironment (TME), IL-6 is among the most expressed cytokines in the inflammatory stroma subtype in patient tumors (17). Moreover, the inflammatory IL-6/STAT3 regulatory circuit, which is maintained by pancreatic stromal cells in the TME, modulates tumor aggressiveness and metastatic properties (36). We therefore analyzed the RNA expression of FACS-purified neoplastic epithelial (EPCAM^+^CD45^–^), immune (EPCAM^–^CD45^+^), and cancer-associated fibroblast–enriched (CAF-enriched) (EPCAM^–^CD45^–^) compartments in 29 human PDAC tumors (Figure 6M). We found a positive correlation between neoplastic *AXL* expression and *IL-6* in the corresponding CAF-enriched population of the identical patient tumors (Figure 6M). However, immune *IL-6* did not show a strong correlation with neoplastic *AXL* in patient tumors (Figure 6M). We also noted that the expression of p-STAT3 regulator gene *WNT10A* and that of *AXL* positively correlated in the neoplastic compartment (Figure 6M). To determine whether ROBO3 or AXL regulated stroma-based microenvironment gene signatures, we analyzed ROBO3-silenced RNA-Seq data in BL PANC1 cells as well as publicly available shAXL RNA-Seq data sets (Figure 6, N and O). Interestingly, gene sets enriched for inflammatory responses, ECM organization, and fibroblast migration were negatively enriched in ROBO3- and AXL-depleted tumor cells (Figure 6, N and O). Together, these results suggest that the ROBO3/AXL regulatory network maintains the IL-6/STAT3 signaling axis in the PDAC TME, contributing to the disease’s aggressiveness.

### Inhibition of the ROBO3/AXL signaling network results in a favorable outcome.

**Figure 7:**
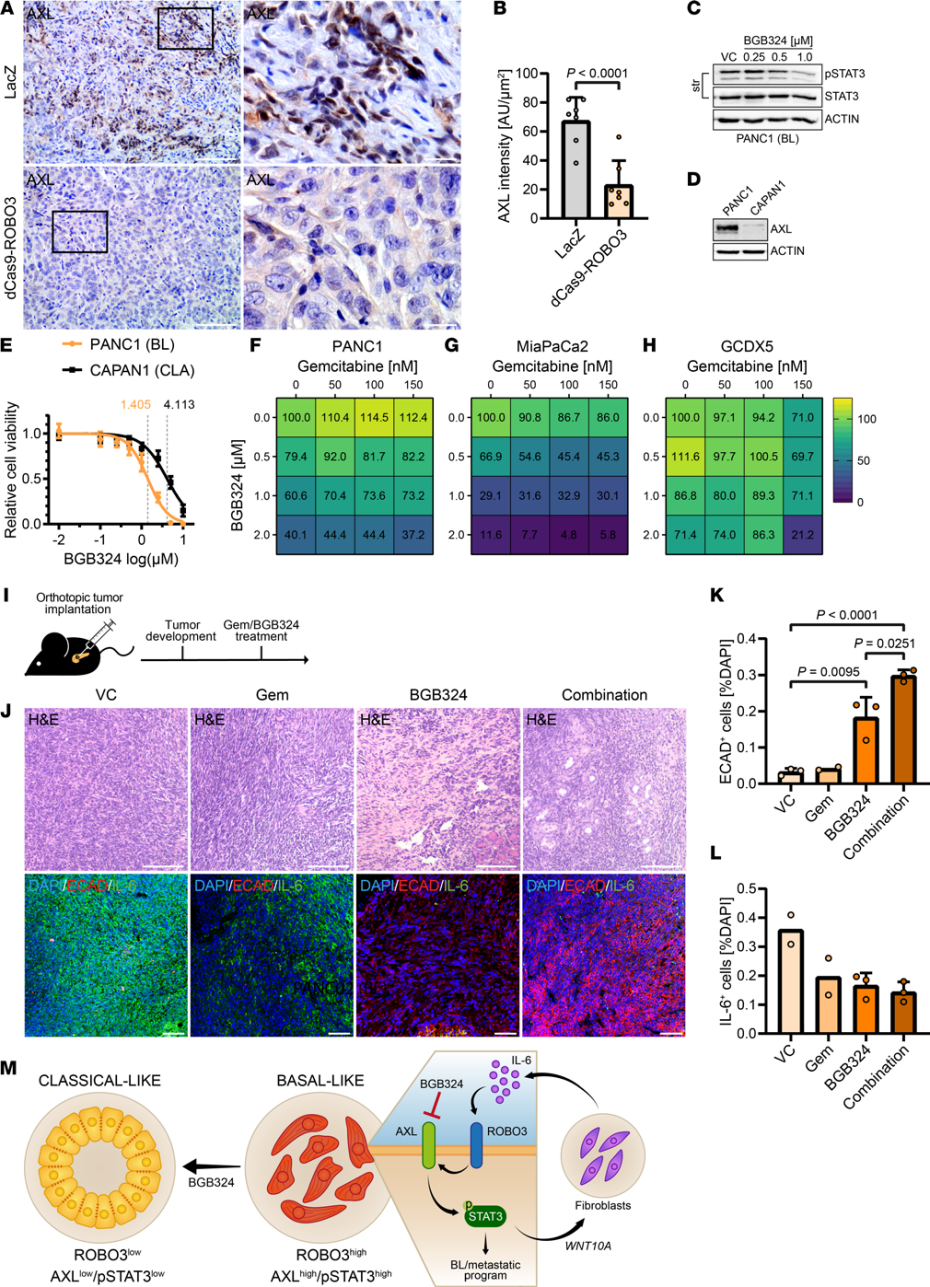
Inhibition of the ROBO3/AXL/p-STAT3 axis leads to a favorable outcome. (**A**) Representative IHC staining for AXL in orthotopic LacZ control or dCas9-ROBO3 PANC1 tumors in NMRI-*Foxn1^nu/nu^* mice. Right: higher magnification of indicated area. Scale bar: 50 μm, magnified area (right panel), 10 μm. (**B**) Quantification of **A**. Scatterplots show intensity, given in AU/μm^2^ as average per animal and means ± SD as bar graphs. Statistical significance determined by Mann-Whitney test. LacZ, *n* = 7; dCas9-ROBO3, *n* = 7. (**C**) Immunoblot for Y705-phosphorylated STAT3 (p-STAT3), total STAT3, and β-actin, in BL PANC1 cells treated with indicated concentrations of BGB324 for 24 hours or vehicle control (VC). (**D**) Immunoblot for AXL and β-actin in BL PANC1 and CLA CAPAN1 cells. (**C** and **D**) Representative of *n* = 3 independent experiments; β-actin as loading control. (**E**) Cell viability of BL PANC1 and CLA CAPAN1 cells after treatment with BGB324 for 18 hours. Respective IC_50_ values are indicated. *n* = 3. (**F**–**H**) Cell viability of BL PANC1 (**F**), MiaPaCa2 (**G**), and GCDX5 (**H**) cells after treatment with indicated concentrations of gemcitabine (Gem) and BGB324 for 24 hours. *n* = 3. (**I**) In vivo experimental design for orthotopic implantation of BL PANC02 cells in immunocompetent C57BL/6 syngeneic mice (35). (**J**) Representative H&E and immunofluorescence (IF) staining for E-cadherin (ECAD) and IL-6 in mice bearing orthotopic PANC02 tumors treated with either Gem, BGB324, or a combination thereof, or VC. (**K** and **L**) Quantification for ECAD (**K**) and IL-6 (**L**) of **J**. Scatterplots show number of ECAD- or IL-6–positive cells (as percentage of DAPI-positive cells) as average per animal, with means ± SD as bar graphs. Statistical significance determined by unpaired Student’s *t* test. (**K**) VC, BGB324, combination, *n* = 3; Gem, *n* = 2. (**L**) VC, Gem, *n* = 2; BGB324, combination, *n* = 3. (**M**) Therapeutic vulnerability of the basal specific hierarchical ROBO3/AXL/p-STAT3 network by BGB324 treatment.

Next, we examined AXL levels in orthotopic tumors derived from the LacZ and dCas9-ROBO3 mouse model. dCas9-ROBO3 animals had significantly reduced metastatic rate compared with LacZ control mice (Figure 3E). Consistent with the view that the ROBO3/AXL regulatory network promoted tumor aggressiveness through the IL-6/STAT3 axis, we observed significantly reduced expression of AXL and p-STAT3 in dCas9-ROBO3 tumors, whereas tumors derived from LacZ showed robust expression of AXL and p-STAT3 (Figure 7, A and B; and Figure 4, E and F). It was evident that ROBO3 mediated PDAC aggressiveness in vitro as well as in vivo; however, pharmacological agents that could target ROBO3 in any cancer type have not been reported to our knowledge. The inhibitor BGB324, which specifically targets AXL tyrosine kinase activity in PDAC (38, 42), is currently being tested in a randomized clinical trial for advanced PDAC (ClinicalTrials.gov NCT03649321). Thus, BGB324 can offer an alternative approach for the treatment of ROBO3-driven aggressive PDAC tumors. Therefore, we first examined the impact of BGB324 on p-STAT3 activity; BGB324 treatment markedly reduced p-STAT3 activity in BL cell lines (Figure 7C and Supplemental Figure 6F). Next, we examined therapeutic efficacy of BGB324 in AXL^hi^ and AXL^lo^ BL and CLA cell lines, respectively (Figure 7D), which showed a marked reduction in proliferation of AXL^hi^ BL cells compared with AXL^lo^ CLA cells (Figure 7E), consistent with the AXL-dependent therapeutic effect of BGB324. We then evaluated the combined therapeutic effect of gemcitabine and BGB324 in vitro; BGB324 alone was effective in reducing the proliferation of both established as well as PDX-derived BL cells in a dose-dependent manner (Figure 7, F–H). Overall, combination of gemcitabine and BGB324 treatment showed an additive to synergistic response in PDAC cells (Figure 7, F–H; and Supplemental Figure 6, G–I). BGB324, in combination with gemcitabine, is known to reduce distant metastases and desmoplastic stromal components in preclinical mouse models (38, 42). To determine whether targeting AXL by BGB324 alone, or in combination with gemcitabine, could reverse BL to a CLA-like phenotype, we utilized a previously reported (38) highly aggressive PANC02-derived C57BL/6 syngeneic orthotopic model (Figure 7I). Combination therapy using BGB324 and gemcitabine (median survival [ms] 48 days) substantially improved survival compared with control (ms 26 days), gemcitabine alone (ms 30 days), or BGB324 alone (ms 28 days) as in a previous study (38). We probed tumor tissues from this study for an established CLA/epithelial differentiation marker E-cadherin (ECAD). In addition, we looked for the expression of IL-6 in the TME of PANC2-derived orthotopic tumors. We found a significant induction of ECAD and reduced expression of IL-6 mainly in the BGB324-alone and combination therapy groups (Figure 7, J–L). Thus, AXL inhibition by BGB324 in combination with gemcitabine can pharmacologically disrupt ROBO3-driven BL subtype aggressiveness and favor acquisition of a CLA-like chemosensitive phenotype in PDAC (Figure 7M).

## Discussion

Comprehensive whole-genome and transcriptome analyses have revealed the presence of 2 well-defined and clinically relevant subtypes in PDAC. The CLA phenotype can be stratified by the expression of epithelial lineage gene signatures, e.g., GATA6, and is generally less aggressive and sufficiently responsive to chemotherapy compared with the BL subtype (16, 20, 30, 43). BL tumors are characterized by early tumor cell invasion, metastasis, and resistance to chemotherapy (14, 16, 17). Recent studies have revealed that the PDAC subtype generation is a reversible process that is largely regulated at the level of transcription, and hence, offers new therapeutic opportunities (27, 37, 44). Here, we examined the role of axon guidance signaling in subtype specificity and focused on ROBO3, which has previously been attributed to a particularly aggressive tumor behavior and poor clinical outcome in patients with PDAC (22, 26). We show that ROBO3 was highly expressed in a greater proportion of PDAC samples, particularly in pancreatic tumors with poor differentiation and a BL phenotype. In addition, we provide strong evidence that ROBO3 receptor signaling was involved in the regulation of subtype-specific gene signatures, which blocked acquisition of a highly aggressive and metastatic BL subtype. Genetic inactivation of ROBO3 blocked the metastatic potential in BL tumors, reduced ascites formation, and resensitized BL tumor cells to gemcitabine treatment.

Mechanistically, ROBO3 controlled BL-specific gene expression, partly through tyrosine kinase AXL-mediated activation of the inflammatory and protumorigenic signaling and transcription factor STAT3. Activation of STAT3 has previously been implicated in the regulation of cell plasticity, tumor cell invasion, and metastases (36, 45–48). For the first time to our knowledge, we provide experimental evidence for the existence of a ROBO3/AXL/p-STAT3 regulatory axis in cancer. We show that (i) a strong correlation exists between ROBO3 expression, AXL, and p-STAT3 in high-grade tumors with BL phenotype; (ii) ROBO3 controls STAT3 Y705 phosphorylation and IL-6–induced p-STAT3 activation and gene signatures in vitro and in vivo; and (iii) AXL tyrosine kinase is a potentially novel and critical player in the ROBO3/p-STAT3 signaling pathway that drives acquisition of the BL PDAC subtype.

Our study contributes to a better understanding of cellular plasticity and regulation of subtype-specific functions in PDAC. We confirm that subtype-specific plasticity is reversibly and dynamically regulated at the transcriptional level. The integrity of the ROBO3/AXL/p-STAT3 regulatory network is essential for a successful signal transduction of inflammatory stimuli, e.g., IL-6, as well as regulation of cellular plasticity and maintenance of the BL PDAC subtype. Accordingly, genetic or pharmacological disruption of the pathway blocks BL subtype functions in favor of the CLA-like phenotypic state. The relevance of STAT3 in cell plasticity and tumor progression is well established; its activation is controlled in a context-dependent manner by multiple cytokines (46, 48, 49). A master inducer of STAT3 activation in tumor and inflammatory cells is IL-6. In PDAC, IL-6–driven phosphorylation of STAT3 at Y705 leads to formation of a mesenchymal phenotype with characteristic features of a highly aggressive phenotypic state (36, 38). Moreover, pharmacological inhibition or genetic inactivation of the IL-6/STAT3 pathway inhibits the invasive capacity of tumor cells, and hence, prevents metastatic colonization in PDAC (36). Our study emphasizes an important role for ROBO3 receptor signaling in STAT3 activation. We show, for the first time to our knowledge, that ROBO3 expression was required for both endogenous and IL-6–induced STAT3 activation, and therefore, loss of ROBO3 caused a dramatic reduction in STAT3 phosphorylation. We also show that in PDAC, IL-6 was preferentially secreted by stromal cells to induce ROBO3/p-STAT3 signaling and formation of a BL phenotype in neoplastic epithelial cells. IL-6–induced activation of the ROBO3/STAT3 pathway in CLA-like tumor cells resulted in the induction of BL-characteristic gene signatures and the acquisition of an EMT-like phenotype. This subtype switch could be prevented almost completely by genetic inactivation of the ROBO3/p-STAT3 signaling pathway. These data support the paradigm of a reversible transcriptional reprograming of cellular plasticity in PDAC, which provides a rationale for novel therapeutic interventions. We have therefore examined the therapeutic potential of pathway disruption by utilizing BGB324, an AXL inhibitor that is currently under clinical investigation in advanced PDAC (38, 42). BGB324 markedly reduced p-STAT3 activity in BL PDAC cells, switched BL tumors toward a more differentiated CLA-like phenotype, and reduced the expression of stromal IL-6, particularly when combined with gemcitabine.

In conclusion, we provide a hierarchical regulatory framework in the BL subtype of PDAC, which underlies EMT, an inflammatory stromal program, and metastatic colonization. We show a likely unique receptor signaling platform that activated p-STAT3, which, in turn, promoted the BL/metastatic phenotypic state in PDAC. Thus, targeting the ROBO3/AXL/p-STAT3 regulatory network may improve response to conventional chemotherapy and offer favorable prognosis in a selective cohort of patients with PDAC.

## Methods

### Preclinical animal studies

The KPC mouse models have been described previously (33). The histopathological characterization of KPC tumors into low-grade or W/M differentiated (G1 and G2), and high-grade or poorly differentiated (G3 and G4), was performed at the University Medical Center Göttingen (UMG). For orthotopic mouse models, 1 × 10^6^ human PDAC cells (CAPAN1 and PANC1) were orthotopically implanted into the pancreas of 10-week-old male NMRI-*Foxn1^nu/nu^* mice (Janvier Labs), as described previously (30). Three weeks after tumor cell implantation, a high-resolution ultrasound was performed to monitor tumor size, as described previously (30, 33). For Figure 7I, this experiment (PANC02-derived C57BL/6 syngeneic orthotopic model; ref. 38) was performed at UT Southwestern Medical Center. For in vivo studies, CRISPR-mediated dCas9-ROBO3 or LacZ control PANC1 cells were orthotopically implanted into the pancreas of 10-week-old male NMRI-*Foxn1^nu/nu^* mice. Mice were sacrificed following body weight loss of more than 20%, at poor overall status, or at experiment endpoint, i.e., 3–5 weeks after detection of a decent sized tumor. Then, tissues were collected for further analysis.

### Establishment of primary tumor cells from PDXs

The PDX mouse model was generated by using surgically resected PDAC specimens from patients. PDAC tumor biopsies were reimplanted in the flanks of immunocompromised mice at the UMG. At 4–5 weeks after xenograft implantation, tumors were isolated and reimplanted for further expansion for at least 3 to 4 generations. The corresponding PDX tumors were harvested, and subsequently, primary tumor cells were isolated, as described previously (44). Primary pancreatic tumor cells (GCDX cells) were maintained in type I collagen–coated (Enzo) plates for 3 to 4 passages. GCDX cells were transferred to normal culture flasks and maintained in keratinocyte-SFM (Life Technologies) and RPMI 1640 medium (Thermo Fisher Scientific) in a 3:1 ratio, supplemented with 2% (v/v) FCS, 1% (v/v) penicillin/streptomycin (P/S) (Sigma), bovine pituitary extract, and EGF.

### Cell culture experiments

Human PDAC cell lines (CAPAN1, CAPAN2, BXPC3, PANC1, and MiaPaCa2) were purchased from ATCC. L3.6 cell line was provided by Daniel D. Biladeau (Division of Oncology Research, Mayo Clinic, Rochester, Minnesota, USA). The CAPAN1 (RRID:CVCL_0237), CAPAN2 (RRID:CVCL_0026), L3.6 (RRID:CVCL_0384), BXPC3 (RRID:CVCL_0186), PANC1 (RRID:CVCL_0480), and MiaPaCa2 (RRID:CVCL_0428) cell lines were authenticated by the German Collection of Microorganisms and Cell Cultures GmbH. The PDAC cell lines were maintained in RPMI 1640 (CAPAN1 and CAPAN2), MEM (L3.6), or DMEM (PANC1 and MiaPaCa2) supplemented with 10% FCS (Thermo Fisher Scientific) and 1% P/S. CRISPR/dCas9-ROBO3–silenced or LacZ control PANC1 or MiaPaCa2 cells were maintained in DMEM supplemented with 10% FCS and 1 μg/mL puromycin (Thermo Fisher Scientific).

### CRISPR/dCas9-mediated manipulation of ROBO3 gene

For the ROBO3 stable knockdown, dCas9 mammalian expression vector and Cas9 sgRNA vector were used. A total of 2 × 10^5^ PDAC cells per well were seeded and then transfected together with dCas9-EGFP vector and the sgRNA-LacZ. Lipofectamine 2000 (Invitrogen) and Opti-MEM (Thermo Fisher Scientific) were used to transfect the corresponding PDAC cells. For 72 hours after transfection, cells were maintained in 1 μg/mL puromycin-containing medium. For the ROBO3 transient overexpression, PDAC cells were transfected with different sgRNA with either dCas9-p300 (D1399Y) control or dCas9-p300 (Core) vectors. Transfection of the PDAC cell line was performed as described above. For vector information and gRNA sequences, see Supplemental Table 1.

### Histopathology, IHC, and IF

Murine and human PDAC tissue specimens were fixed in 4% (v/v) paraformaldehyde and embedded in paraffin blocks. Tissues were sectioned at a thickness of 4–5 μm. H&E, IHC, and IF staining were performed as described previously (30, 44) (Supplemental Table 2). Laser confocal microscopy (Olympus FluoView 1000) was used for the IF image capture. All the acquired images were quantified either by intensity-based measurement (given as AU) or by manual counting using ImageJ Fiji software (NIH) as described previously (50). For IF in cells, PDAC cells were seeded on glass coverslips, and after 24 hours, cells were treated with either IL-6 (50 ng/mL) or vehicle control for 24 hours or 48 hours. Cells were then fixed in 4% (v/v) paraformaldehyde for 15 minutes and washed 3 times in phosphate buffer. The coverslips were removed and placed for blocking (2% normal goat serum) and antibody incubation, as described above.

### Tile image

VS120 virtual slide microscope (Olympus) was used for whole-slide scans of murine and human PDAC tissues. cellSens Dimension software (Olympus) was used for image analyses.

### Human PDAC TMA analysis and quantification

In total, 62 matched PDAC TMA tissues were analyzed, with 1 to 3 tissue cores per case being evaluated. PDAC TMA was stained by IHC with p-STAT3, STAT3, and ROBO3 antibodies (Supplemental Table 2), as described above. TMA evaluation and data acquisition were performed as described previously (45).

### siRNA transfection

PDAC cells were seeded in 6-well plates and transfected with either 20 nM siRNA against ROBO3, AXL, and STAT3, or control siRNA (see Supplemental Table 1). siLentFect lipid reagent (Bio-Rad, 170-3362) or Lipofectamine 2000 transfection reagents were used as per manufacturers’ instructions. After 48–72 hours, proteins and RNA were extracted for the analysis.

### qRT-PCR

Cells were washed with PBS and collected in TRIzol (Invitrogen). RNA was isolated using phenol-chloroform extraction method. RNA quality and concentration were determined spectrophotometrically (Intas NanoPhotometer). cDNA synthesis was carried out using iScript cDNA synthesis kit (Bio-Rad). qRT-PCR was performed in triplicates using SYBR Green (Bio-Rad) with StepOne Plus Real-Time PCR System (Applied Biosystems). qRT-PCR primers used in this study are listed in Supplemental Table 1.

### RNA-Seq

ROBO3 siRNA-mediated silencing in PANC1 cells was performed as follows. Cells were lysed in TRIzol for total RNA isolation (3 biological replicates). After RNA quality validation via agarose gel electrophoresis, 500 ng of total RNA was used for cDNA library preparation using TruSeq RNA Library Prep kits (Illumina, RS-122-2001; RS-122-2002), as per manufacturer’s instructions. Qubit dsDNA high-sensitivity assay was utilized for cDNA concentration measurement (Thermo Fisher Scientific, Q32854). Bioanalyzer high-sensitivity DNA analysis (Agilent, 5067-4626) was used for DNA fragment size measurement prior to sequencing (single-end 50 bp) on a HiSeq2000 (Illumina), performed at the NGS Integrative Genomics Core Unit at UMG.

#### Data analysis.

Sequencing data were processed in the GALAXY environment provided by the Gesellschaft für wissenschaftliche Datenverarbeitung mbH Göttingen; the raw read quality was examined using FastQC v0.71. STAR v2.5.2b (51) was utilized to align sequence reads to the hg38 human reference genome. Next, the featureCounts tool (ref. 52; v1.6.0.2) was used to estimate reads per gene, and differential gene expression was calculated with DESeq2 v2.11.40.1 (53). Gene signature enrichment analyses were performed using the GSEA tool. The BL-A and BL-B signatures were defined as described previously (14) and are detailed in Supplemental Table 3.

### Patient expression data of human epithelial and immune PDAC cells

Transcriptome analysis of resected human PDAC epithelial, immune, and cancer-associated fibroblasts was performed at the Department of General, Visceral and Transplantation Surgery, University Hospital Heidelberg, using material of patients who underwent partial pancreatoduodenectomy. Epithelial, immune, and cancer-associated fibroblast populations were isolated via flow cytometry after dead cell exclusion using propidium iodide. RNA extraction, library preparation, and RNA-Seq were performed as described previously (54).

### Multiplex profiling of protein tyrosine kinase substrates

Whole-cell lysates were extracted from transiently ROBO3-silenced or control siRNA-transfected PDAC cells, as described above. A total of 5 μg of protein lysates was prepared in 40 μL final volume of kinase master mix containing the kinase assay buffer (50 mM Tris-HCl pH 7.5, 10 mM MgCl_2_, 1 mM EGTA, 2 mM dithiothreitol, 0.01% Brij 35, 1 mg/mL BSA, 12.5 μg/mL FITC-labeled PY-20 antibody, and 0.4 mM ATP) included in the PTK reagent kit (PamGene, 32112), according to the manufacturer’s instructions. The software Evolve (PamGene) was used for initial sample and array processing as well as image capture. Sample and array annotation, image gridding, quality control, and phosphorylation signal quantitation were performed using the software package BioNavigator (version 6.2; PamGene). Upstream kinase prediction was based on tyrosine phosphorylation patterns and automatically calculated by default using the BioNavigator analysis software tool, as described previously (55, 56).

### 3D invasion assay and immunofluorescence

The bottom of Transwell inserts (8 μm pores) was coated with a cocktail of 50 μL collagen type I (Enzo) diluted in 0.1 M HCl. After collagen solidification, cells (1 × 10^5^) were mixed with Matrigel (Th. Geyer GmbH & Co KG, 1025615) and seeded into the Transwell inserts. Cells were incubated for at least 30 minutes at 37°C. Next, culture medium was poured into the inserts (250 μL) and in the 24-well plates (750 μL) and incubated for 48 hours at 37°C. Cotton swabs were then used to remove the Matrigel from the inserts. The invaded cells at the bottom of the insert were fixed for IF experiments and stained. A confocal microscope (Olympus FluoView 1000) was used to capture images of invaded cells. Total number of invading cells per 20× original magnification F.o.V. was counted either manually or by ImageJ software (50) and averaged per independent insert. For each biological replicate, 2 independent inserts per condition were evaluated. Antibodies are detailed in Supplemental Table 2.

### Flow cytometry analysis

A total of 1 × 10^6^ cells were used to measure cell surface expression of IL-6 in PDAC cell lines. For the flow cytometric analysis, cells were transferred into a 96-well plate, followed by centrifugation at 300*g* for 5 minutes at room temperature. Cells were washed in FACS buffer [10% knockout serum in PBS (-Ca/-Mg)], then stained with anti–IL-6 or control IgG antibodies for 30 minutes at room temperature. Prior to FACS analysis, cells were gently washed 2 times with FACS buffer. For IL-6 analysis, a 561 nm laser (BD Biosciences) was used to detect the signal. The antibodies used in this study are listed in Supplemental Table 2.

### Cell viability assay

The CellTiter-Glo Luminescent Cell Viability Assay (Promega) was performed to quantify the viable cells, according to the manufacturer’s instructions. For combination therapy experiments, 2500 cells/well were seeded in 96-well plates and treated with gemcitabine, BGB324, or in combination with different doses. For chemotherapy response studies after AXL silencing, 100,000 cells were seeded in 6-well plates and transfected with AXL-targeting or control siRNA as detailed above. After 24 hours, silenced cells were trypsinized, counted, and seeded for gemcitabine or control treatment.

After time points indicated in the figures, cells were harvested and incubated with CellTiter-Glo assay. LUmo Luminometer was utilized to measure the luminescence.

To determine synergies, the average dose responses were used as input for the SynergyFinder 2.0 tool (http://synergyfinder.fimm.fi, ref. 57). Synergy scores were calculated by the zero interaction potency model and visualized in 3D surface plots. Summary synergy scores over all concentrations are indicated in the figures.

### Western blot and co-immunoprecipitation analysis

PDAC cells were lysed in lysis buffer supplemented with cOmplete protease inhibitor (Roche; 25× stock), PMSF, and sodium orthovanadate. Protein samples were separated on 10%–15% (v/v) SDS-polyacrylamide gels. After blotting, membranes were blocked with 5% milk powder (*w/v*) in TBS Tween buffer and incubated with antibodies as listed in Supplemental Table 2. Intas ChemoCam Imager and ECL substrate (Bio-Rad) were used to develop the membranes. If indicated in the figures, membranes were stripped using NaOH, blocked, and reblotted with another primary antibody as detailed above. The co-immunoprecipitation experiment was performed as described previously (45).

### Data availability and analysis

RNA-Seq data generated for this study are available at ArrayExpress database under accession number E-MTAB-11476.

#### PDAC patient expression data.

Microarray gene expression data were downloaded from ArrayExpress database with accession number E-MTAB-6134 (17). In the case of multiple probes per gene, the probe with highest average values across all patients was used for correlation analysis. RNA-Seq data of FACS-sorted epithelial patient tumor are available at European Genome-phenome Archive under accession code EGAS00001004660 (58).

#### Effect size meta-analysis.

The effect size (i.e., log_2_ fold-change) and its standard error for ROBO3 and AXL, respectively, were extracted from the respective genome-wide differential expression analysis of 5 studies where global expression in CLA/pancreatic progenitor tumors was compared with BL/squamous/quasi-mesenchymal tumors. Meta-analysis was carried out using the metafor R package (59). Both random and fixed effect models were fit using the rma function (method = “REML” and method = “FE”). Q-test and I2 indicate measures of interstudy heterogeneity.

#### Human PDAC cohorts.

Definitions for this study’s cohorts and data sets are the following: PID, Pathway Interaction Database; KEGG, Kyoto Encyclopedia of Genes and Genomes; COLLISSON, Collisson et al. (29); CUMC, Columbia University Medical Center (28); UNC, University of North Carolina at Chapel Hill (18); TCGA, The Cancer Genome Atlas; and ICGC, International Cancer Genome Consortium. The following human PDAC cohorts were used to study differential expression of ROBO3 and AXL, respectively, between BL/squamous/quasi-mesenchymal and CLA/progenitor tumors. For the CUMC cohort, raw count data for 60 epithelial PDAC samples were retrieved from Gene Expression Omnibus (GEO) GSE93326 (28); for the TCGA-PAAD cohort, raw count data were retrieved from the NIH National Cancer Institute GDC Data Portal for 149 patients as described previously (15). In each case, the variance was stabilized by fitting the dispersion to a negative binomial distribution as implemented in the DESeq2 R package (53). For the ICGC-PACA-AU cohort described previously (10), normalized gene expression data for 96 patients were provided by the authors in the original publication (10). Microarray data of primary PDAC specimens from Collisson et al. (*n* = 27) and Moffitt et al. (*n* = 125) were retrieved from GSE17891 (29) and GSE71729 (18), respectively. Only the following classes were used for differential gene expression analysis in the respective cohorts: CLA or pancreatic progenitor, respectively, versus BL, squamous, or quasi-mesenchymal, respectively.

For gene set overrepresentation analysis in G3 versus G1 PDAC patient tumors of the TCGA data set, differential expression analysis was performed on the R2 platform (Genomics Analysis and Visualization Platform, http://r2.amc.nl) using the default parameters. Significantly different genes were subset by fold-change into genes corresponding to either G3 or G1, and overrepresentation analysis was performed using the enricher function of the clusterProfiler package version 3.18.1 in R (60).

#### Publicly available shAXL sequencing data.

RNA-Seq data of shAXL and shCtrl in lung cancer cells were downloaded from GEO database with accession number GSE128417. Differential expression analysis was performed using DESeq2 version 1.30.1 in R version 4.0.5 (53). For GSEA, clusterProfiler version 3.18.1 was utilized.

### Statistics

GraphPad Prism version 8.0.2 software was utilized for statistical analysis. The data for comparison of 2 groups were analyzed by a 2-tailed Mann-Whitney or Student’s *t* test. Statistical analysis for survival data was performed by a log-rank test. Gene expression correlation analysis for FACS-sorted patient RNA-Seq was analyzed by Spearman’s rank correlation. Pearson’s correlation coefficient was performed for publicly available gene expression microarray data using a 2-tailed *P* value. Results were considered significant with a *P* value below 0.05, as indicated in the figures.

### Study approval

Animal experiments were approved and performed according to the guidelines of the Central Animal Facility at the UMG (permission no. 5/2057, 14/1634, 18/2953) and the Ruhr University Bochum (Bochum, Germany; permission no. 8.87-50.10.32.09.018).

Generation of the PDX mouse model was approved by the ethics committee of the UMG (permission no. 70112108) and Ruhr University Bochum (permission no. 3534-9, 3841-10, 16-5792).

The PANC02-derived C57BL/6 syngeneic orthotopic model was performed in accordance with the Institutional Animal Care and Use Committee at UT Southwestern Medical Center (38).

PDAC patient TMA tissues were obtained from the Department of Pathology, UMG, following the ethical approval of the institute (70112108).

FACS of PDAC resection material was approved as part of the HIPO-project (case number S-206/2011 and EPZ-Biobank Ethic Vote 301/2001) approved by the ethical committee of the University Hospital Heidelberg, Heidelberg, Germany (58).

This study was conducted in accordance with the Declaration of Helsinki; written informed consent was collected from all participating patients.

## Author contributions

The study design, data analysis, and data interpretation were performed by NK, LK, FW, and SKS. The orthotopic mouse models, CRISPR/dCas9, and histology were performed by NK, MT, SC, FP, and XX. LK and FW conducted RNA-Seq and bioinformatics analyses. FACS-sorted RNA-Seq analysis and bioinformatics analysis of PDAC patients were performed by EE; data interpretation was performed by both EE and AT. Meta-analysis and data interpretation of publicly available human PDAC specimens were performed by LK and HCM. Multiplex profiling of protein tyrosine kinase was performed by SK and NK; HB, MB, and NK analyzed the human PDAC TMA tissue. EH, AN, PS, UK, and VE helped with pathological examination of human and murine specimens, experimental design, and data interpretation. RAB provided PANC02 tumor tissues and aided us with the data analysis. SKS supervised the project, interpreted the data, and wrote the manuscript. UK and VE edited the manuscript. All authors read and approved the manuscript.

## Supplementary Material

Supplemental data

## Version 1 08/22/2022

Electronic publication
