# Supplementary material for: Axon guidance receptor ROBO3 modulates subtype identity and prognosis via AXL-associated inflammatory network in pancreatic cancer
Source: JCI Insight. 2022 Aug 22;7(16):e154475. doi: 10.1172/jci.insight.154475 (PMC9462476; doi:10.1172/jci.insight.154475)
Supplement: Supplemental data [file jciinsight-7-154475-s040.pdf]

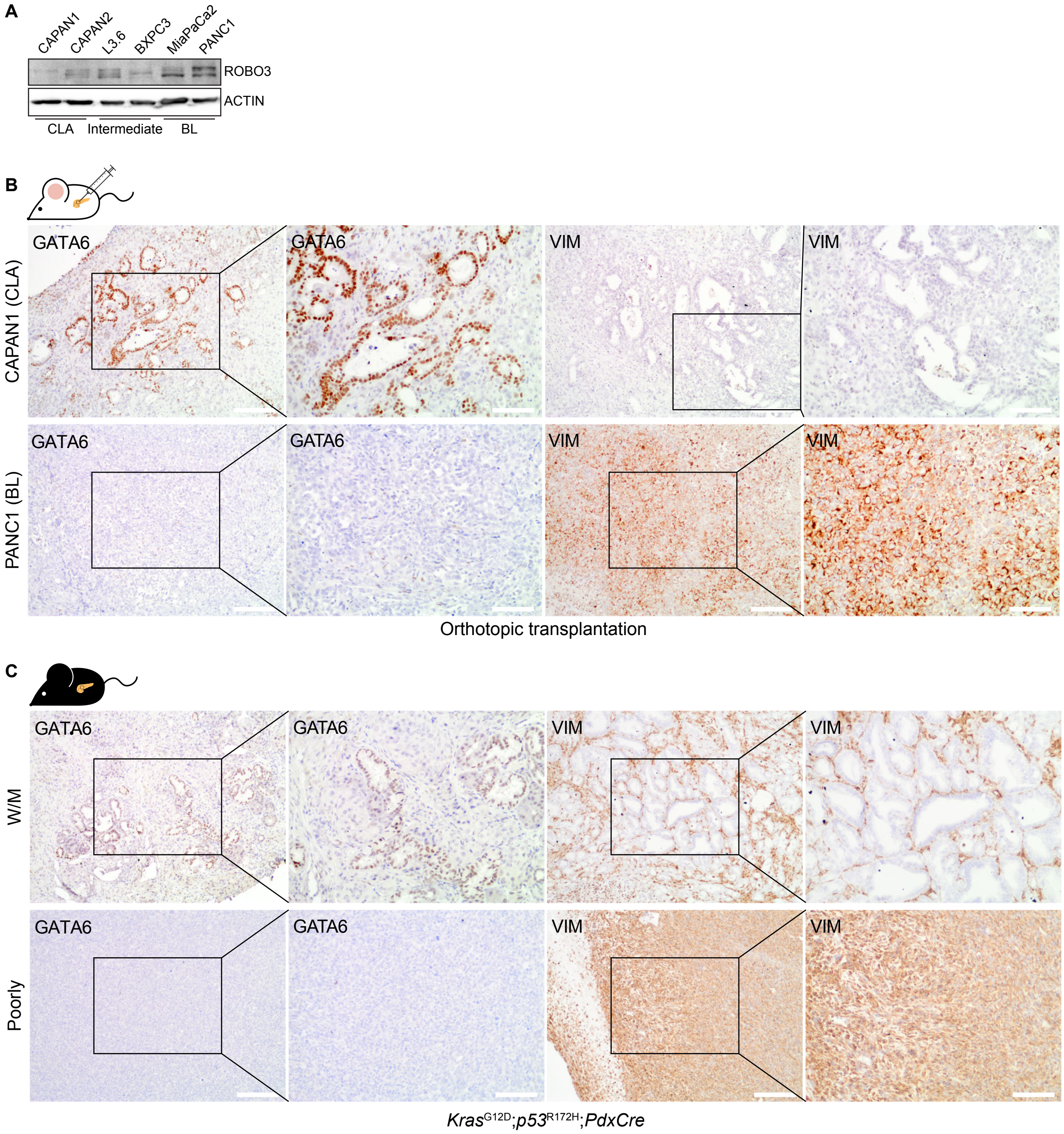

Supplemental Figure 1. PDAC subtype-dependent expression of ROBO3. (**A**) Immunoblot analysis for ROBO3 and β-actin as loading control in CLA (CAPAN1, CAPAN2), intermediate (L3.6, BXPC3) and BL (MiaPaCa2, PANC1) PDAC cell lines. Representative of n=3. (**B**,**C**) Representative IHC staining for GATA6 and vimentin (VIM) in orthotopically implanted CLA (CAPAN1) and BL (PANC1) cell lines in the pancreas of NMRI-*Foxn1*$^{nu/nu}$ mice (**B**), as well as in *Kras*^{G12D};*p53*^{R172H};*Cre* (KPC) tumors, histopathologically graded by expert pathologists (**C**). Higher magnification of the indicated areas are shown. Scale bar: 200 µm; for magnified area (right panels), 50 µm. (**B**) CLA, n=3; BL, n=3. (**C**) W/M, n=4; poorly, n=4.

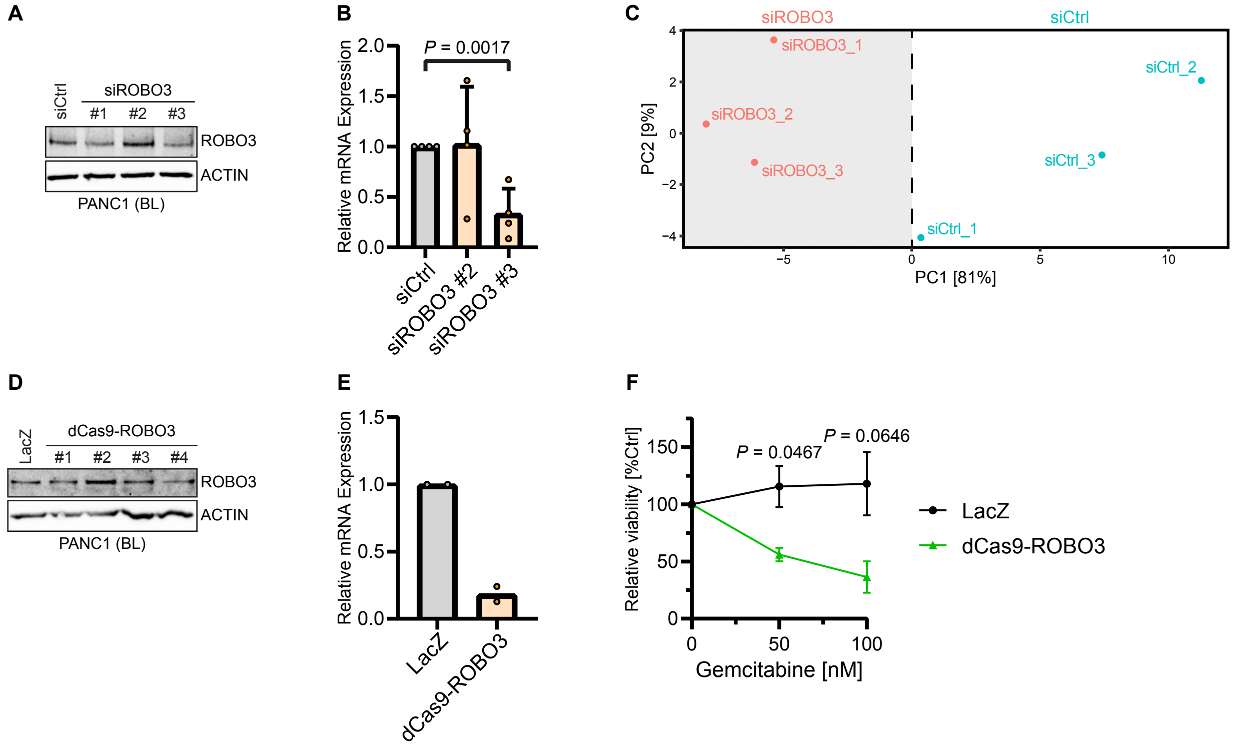

Supplemental Figure 2. Depletion of ROBO3 in BL PDAC. (**A**) Immunoblot analysis for ROBO3 and β-actin in BL (PANC1) cells transfected with different sequences of ROBO3-targeting (siROBO3) or control siRNA (siCtrl). Representative of n=3 independent experiments. Sequence #3 was used for all other experiments in this study. (**B**) RT-qPCR analysis of ROBO3 in BL PANC1 cells transfected with siROBO3 or siCtrl. Results show average relative quantification (to control treatment) ± SD. n=4. (**C**) PCA plot of RNA-seq data performed on BL PANC1 cells transfected with siROBO3 or siCtrl. n=3. (**D**) Immunoblot analysis for ROBO3 and β-actin, in BL PANC1 cells with CRISPR/dCas9-mediated knockdown of ROBO3 using different sgRNA-sequences or LacZ-transfected control cells. sgRNA sequence #4 was used for all other experiments in this study. (**E**) RT-qPCR analysis of ROBO3 in LacZ control and dCas9-ROBO3 PANC1 cells. Results show average relative quantification (to control treatment) ± SD. n=2. (**F**) Cell viability of LacZ control and dCas9-ROBO3 PANC1 cells after treatment with the indicated concentrations of gemcitabine for 72h. n=4. (**B**,**F**) Significance was determined by an unpaired Student's t-test.

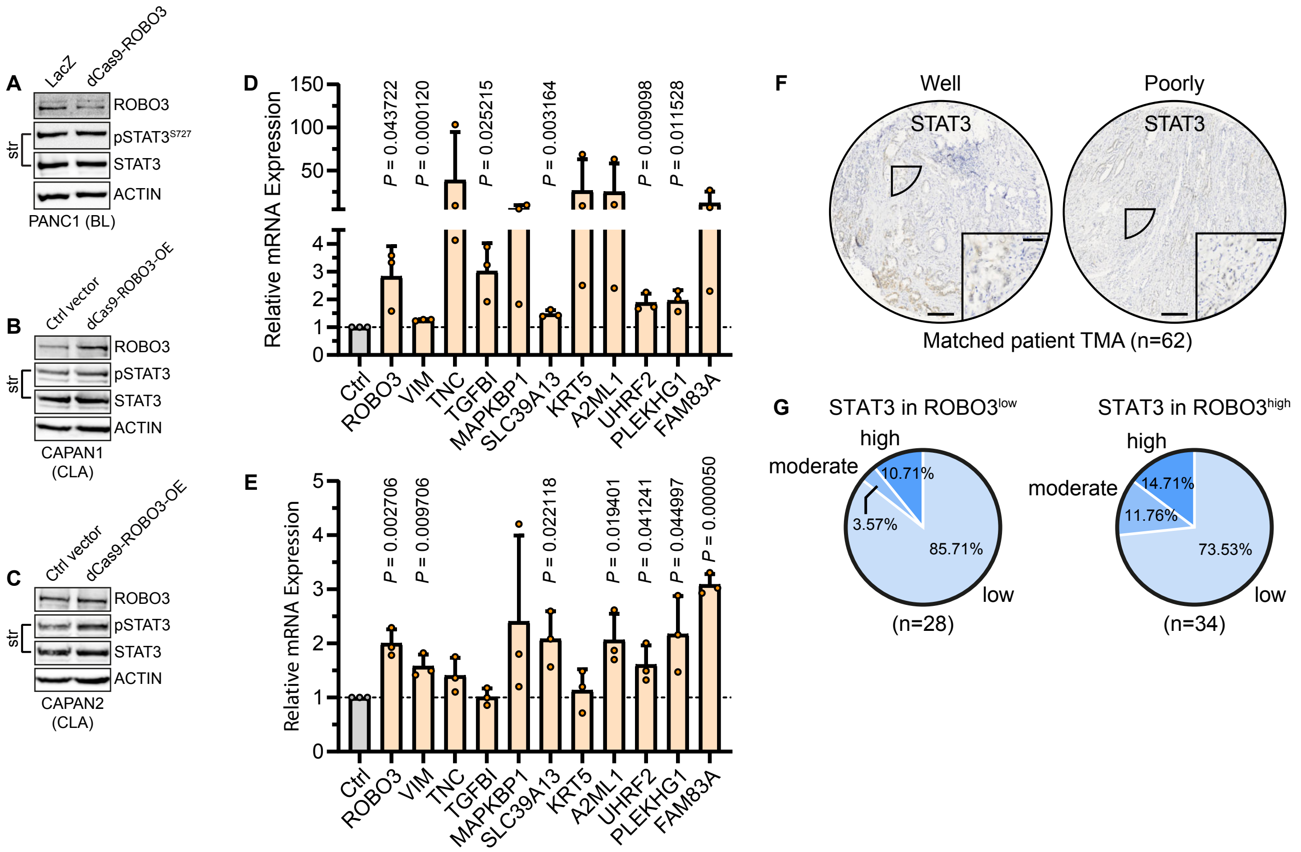

Supplemental Figure 3. ROBO3-dependent pSTAT3 axis. (**A**) Immunoblot analysis for ROBO3, S727-phosphorylated (pSTAT3^{S727}) and total STAT3, as well as β-actin as loading control, in LacZ control and dCas9-ROBO3 PANC1 cells. (**B**,**C**) Immunoblot analysis for ROBO3, Y705-phosphorylated (pSTAT3) and total STAT3, as well as β-actin as loading control, in control vector and dCas9-mediated ROBO3 overexpressing CAPAN1 (**B**) and CAPAN2 (**C**) cells. (**A**-**C**) Representative of n=3 independent experiments. (**D**,**E**) RT-qPCR analysis of selected BL genes, based on the published data of Collisson, Moffitt and Bailey, in control vector and dCas9-mediated ROBO3 overexpressing CAPAN1 (**D**) and CAPAN2 (**E**) cells. Results show average relative quantification (to control vector) ± SD. Significance was determined by an unpaired Student's t-test. n=3. (**F**) Representative IHC staining for STAT3 of tumor microarray (TMA) spots of primary PDAC tissue derived from 62 human PDAC patient resection tissue. Scale bar: 200 µm; for magnified area, 50 µm. (**G**) Evaluation of STAT3 IHC immunoreactive scores (IRS, scale 0-12) of TMA spots in ROBO3^{low} (left; ROBO3 intensity <2, n=28) and ROBO3^{high} (right; ROBO3 intensity ≥2, n=34) patients. Low, IRS<6; moderate, 6≤IRS<8; high, IRS ≥8. Histopathological grading performed by expert pathologists. 1-3 TMA spots were evaluated and averaged per patient. n=62.

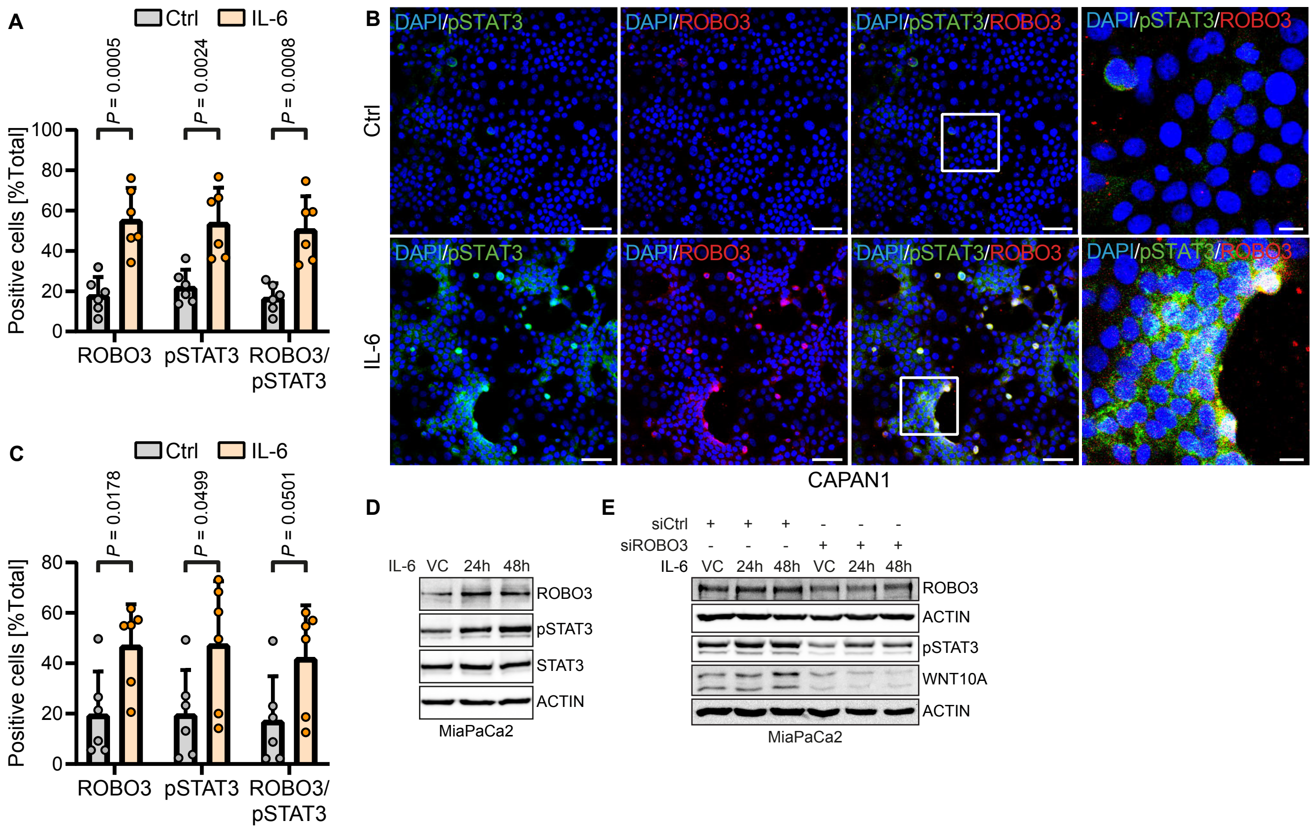

Supplemental Figure 4. IL-6 induces co-expression of pSTAT3^{Y705} and ROBO3. (**A**) Quantification of IF staining for Y705-phosphorylated STAT3 (pSTAT3) and ROBO3 following IL-6 treatment for 48 h or control treatment in basal-like (BL) PANC1 cells. Scatter plots show average number of ROBO3$^+$, pSTAT3$^+$ or double-positive cells as well as means ± SD as bar graphs. Statistical significance was determined by an unpaired Student's t-test. n=6. (**B**) Representative IF staining for pSTAT3 and ROBO3 following IL-6 treatment for 48 h or control treatment in classical (CLA) CAPAN1 cells. Scale bar: 50 µm; for magnified area (right panel), 10 µm. (**C**) Quantification of (**B**), as in (**A**). n=6. (**D**) Immunoblot analysis for ROBO3, pSTAT3, STAT3, as well as β-actin as loading control, in BL MiaPaCa2 cells following IL-6 treatment for 24 and 48 h or VC. (**E**) Immunoblot analysis for ROBO3, pSTAT3, STAT3, WNT10A, as well as β-actin as loading control, in BL MiaPaCa2 cells transfected with ROBO3-targeting (siROBO3) or control siRNA (siCtrl), additionally treated with IL-6 for 24 and 48 h or VC. (**D**,**E**) Representative of n=3 independent experiments.

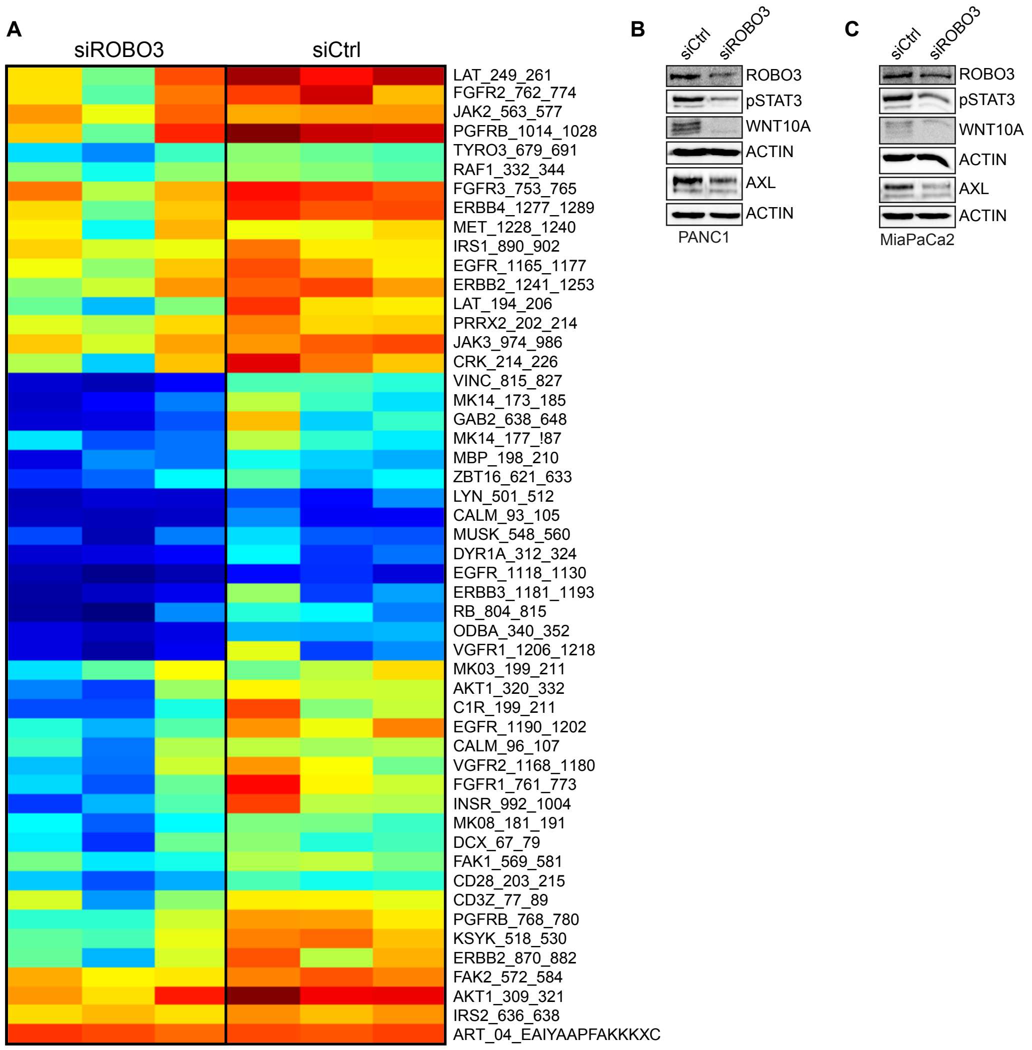

Supplemental Figure 5. ROBO3 activates STAT3 phosphorylation via AXL. (**A**) Differential kinase activity of significant peptide signatures in PANC1 cells upon siROBO3 or siCtrl. Represented in the heatmap are the individual intensities of phosphorylation of all quality-controlled peptides which serve as substrates for tyrosine kinases on PTK chips. Representative of n=3 independent experiments. (**B**,**C**) Immunoblot analysis for ROBO3, AXL, Y705-phosphorylated STAT3 (pSTAT3), WNT10A, as well as β-actin as loading control, in BL PANC1 (**B**) and MiaPaCa2 (**C**) cells transfected with ROBO3-targeting (siROBO3) or control siRNA (siCtrl).

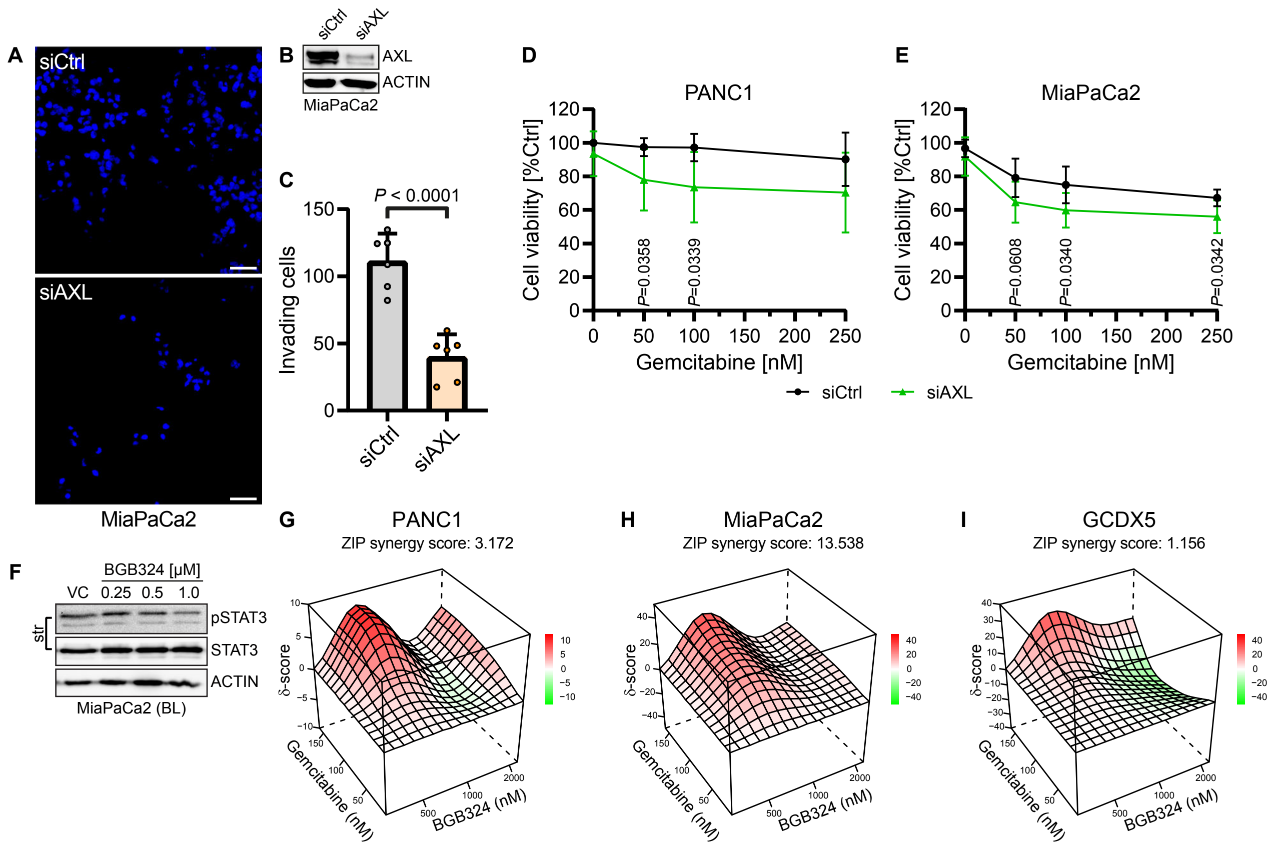

Supplemental Figure 6. AXL silencing leads to reduced aggressiveness in BL cells. (**A**-**C**) Trans-well invasion assay of basal-like (BL) MiaPaCa2 cells transfected with AXL-targeting (siAXL #2) or control siRNA (siCtrl). (**A**) Representative DAPI staining of invaded cells. Scale bar: 50 µm. (**B**) Immunoblot analysis for AXL and β-actin as loading control. Representative of n=3 independent experiments. (**C**) Quantification of (**A**). Scatter plots show average counts as well as means ± SD as bar graphs. Statistical significance was determined by an unpaired Student's t-test. n=6. (**D**,**E**) Cell viability of BL PANC1 (**D**) and MiaPaCa2 (**E**) cells transfected with siAXL (#2) or siCtrl and subsequently treated with the indicated concentrations of gemcitabine for 48 h. n=6. (**F**) Immunoblot analysis for Y705-phosphorylated (pSTAT3) and total STAT3, as well as β-actin as loading control, in BL MiaPaCa2 cells treated with the indicated concentrations of BGB324 for 24 h or vehicle control (VC). Representative of n=3 independent experiments. (**G**-**I**) Synergy plots of PANC1 (**G**), MiaPaCa2 (**H**) and GCDX5 (**I**) cells treated in combination with the indicated concentrations of gemcitabine and BGB324. n=3.

Supplemental Table 1. qRT-PCR primers, siRNAs and constructs

Target		Sequence	Application	Species	Company	Assay-ID
**A2ML1**	Forward	CTAGGAATGTTGGCCCTATCACC	RT-PCR	human	Sigma Aldrich	
	Reverse	CCAAACAAACCTTCTGAACGGAG	RT-PCR	human	Sigma Aldrich	
**AGR2**	Forward	GTCAGCATTCTTGCTCCTTGT	RT-PCR	human	Sigma Aldrich	
	Reverse	GGGTCGAGAGTCCTTTGTGTC	RT-PCR	human	Sigma Aldrich	
**ALDH1B**	Forward	CCCATTCTGAACCCAGACATC	RT-PCR	human	Sigma Aldrich	
	Reverse	AATGACCTCCCCGGTGGTA	RT-PCR	human	Sigma Aldrich	
**CAPN8**	Forward	TGGCTCCAACCAAAACGCTT	RT-PCR	human	Sigma Aldrich	
	Reverse	CCTGGTCCAAGATCCTTGTAGC	RT-PCR	human	Sigma Aldrich	
**CDC42**	Forward	CCATCGGAATATGTACCGACTG	RT-PCR	human	Sigma Aldrich	
	Reverse	CTCAGCGGTCGTAATCTGTCA	RT-PCR	human	Sigma Aldrich	
**cMET**	Forward	AGCAATGGGGAGTGTAAAGAGG	RT-PCR	human	Sigma Aldrich	
	Reverse	CCCAGTCTTGTACTCAGCAAC	RT-PCR	human	Sigma Aldrich	
**ERBB3**	Forward	GGTGATGGGGAACCTTGAGAT	RT-PCR	human	Sigma Aldrich	
	Reverse	CTGTCACTTCTCGAATCCACTG	RT-PCR	human	Sigma Aldrich	
**FAM83A**	Forward	GGCCCTAAGGGACTGGACT	RT-PCR	human	Sigma Aldrich	
	Reverse	CACAGTGGCGCTGGATTTTT	RT-PCR	human	Sigma Aldrich	
**KRT5**	Forward	CCAAGGTTGATGCACTGATGG	RT-PCR	human	Sigma Aldrich	
	Reverse	TGTCAGAGACATGCGTCTGC	RT-PCR	human	Sigma Aldrich	
**MAPKBP1**	Forward	CTGTGGAAGGGTCAACCATTAC	RT-PCR	human	Sigma Aldrich	
	Reverse	GTCCTCTCGTCGGTTTCCTG	RT-PCR	human	Sigma Aldrich	
**Nestin**	Forward	CTGCTACCCTTGAGACACCTG	RT-PCR	human	Sigma Aldrich	
	Reverse	GGGCTCTGATCTCTGCATCTAC	RT-PCR	human	Sigma Aldrich	
**PLEKHG1**	Forward	CTGCACCTGGACTTGACAG	RT-PCR	human	Sigma Aldrich	
	Reverse	CCAACAGCAGATCCGTGAAGA	RT-PCR	human	Sigma Aldrich	
**ROBO3**	Forward	GTAGGACCGGAGGACGCTAT	RT-PCR	human	Sigma Aldrich	
	Reverse	CCCCGTTCTTGTACCACTCA	RT-PCR	human	Sigma Aldrich	
**SLC39A13**	Forward	TCAGCGGCTACCTCAACCT	RT-PCR	human	Sigma Aldrich	
	Reverse	AGGAGCCCGATCTTCTTGCT	RT-PCR	human	Sigma Aldrich	
**SOX2**	Forward	GCCGAGTGGAAACTTTTGTCG	RT-PCR	human	Sigma Aldrich	
	Reverse	GGCAGCGTGTACTTATCCTTCT	RT-PCR	human	Sigma Aldrich	
**TGFBI**	Forward	CTGTGGAAGGGTCAACCATTAC	RT-PCR	human	Sigma Aldrich	
	Reverse	GTCCTCTCGTCGGTTTCCTG	RT-PCR	human	Sigma Aldrich	
**TNC**	Forward	TCCCAGTGTTCGGTGGATCT	RT-PCR	human	Sigma Aldrich	
	Reverse	TTGATGCGATGTGTGAAGACA	RT-PCR	human	Sigma Aldrich	
**UHRF2**	Forward	GGCACATCTACACAGATTGAGG	RT-PCR	human	Sigma Aldrich	
	Reverse	CAAGGCCGACATCTCTGGC	RT-PCR	human	Sigma Aldrich	
**XS13**	Forward	TGGGCAAGAACACCATGATG	RT-PCR	human	Sigma Aldrich	
	Reverse	AGTTTCTCCAGAGCTGGGTTGT	RT-PCR	human	Sigma Aldrich	
**AXL**		#1	siRNA #1	human	ThermoFisher Scientific	s1846
**AXL**		#2	siRNA #2	human	ThermoFisher Scientific	s1847
**ROBO3**		#1	siRNA #1	human	ThermoFisher Scientific	241788
**ROBO3**		#2	siRNA #2	human	ThermoFisher Scientific	s34572
**ROBO3**		#3	siRNA #3	human	ThermoFisher Scientific	29905
**ROBO3 sgRNA**	#1	GGGACTCCGGCACTAGGGGG	CRISPR-dCas9	human		
**ROBO3 sgRNA**	#2	CCCCTAGTGCCGGAGTCCCC	CRISPR-dCas9	human		
**ROBO3 sgRNA**	#3	TTCTCTCCACCCCCTAGTGC	CRISPR-dCas9	human		
**ROBO3 sgRNA**	#4	CCACGGTGCCGCTCTCCTGC	CRISPR-dCas9	human		
**ROBO3 sgRNA**	#1	TCTAGTGTCCTTACGGCCCT	dCas9-p300	human		
**ROBO3 sgRNA**	#2	GCTCGGATTTATGTCTTCCC	dCas9-p300	human		
**ROBO3 sgRNA**	#3	CTTCTGCCCAACTTTAGAAC	dCas9-p300	human		
**pSLQ1658-dCas9-EGFP**			CRISPR-dCas9	human	Addgene	#51023
**Cas9 sgRNA vector**			CRISPR-dCas9	human	Addgene	#68463
**dCas9-p300 Core vector**			CRISPR-dCas9	human	Addgene	#61357

**dCas9-p300 Core(D1399 Y) vector**	CRISPR-dCas9	human	Addgene	#61358
**pSPgRNA vector**	CRISPR-dCas9	human	Addgene	#47108

Supplemental Table 2. Antibodies

Name	Company	Catalog	Dilution
Anti-AXL antibody	Cell Signaling Technology	8661	1:1000 (WB), 1:100 (IHC), 1:200 (IP)
HRP-linked β-Actin	Sigma-Aldrich	A3854	1:40000 (WB)
Anti-pSTAT3 antibody	Cell Signaling Technology	9145	1:1000 (WB), 1:100 (IF; IHC)
Anti-pSTAT3 antibody	Abcam	ab76315	1:1000 (WB)
Anti-ROBO3 antibody	Abcam	ab77261	1:1000 (WB)
Anti-ROBO3 antibody	R & D Systems	AF3076	1:1000 (WB), 1:50 (IF), 1:100 (IHC)
Anti-STAT3 antibody	Cell Signaling Technology	9139	1:1000 (WB), 1:500 (IHC)
Anti-GATA6 antibody	R D Systems	AF1700	1:350 (IHC)
Anti-VIM antibody	Abcam	ab92547	1:200 (IHC) (for murine)
Anti-VIM antibody	Pharmingen	550513	1:150 (IHC) (for human)
Anti-WNT10A antibody	Sigma-Aldrich	ABS456	1:1000 (WB)
Anti-CD45 antibody	Miltenyi Biotec	5B1	1:10 (FC)
Anti-CD326 (EpCAM) antibody	Miltenyi Biotec	HEA-125	1:10 (FC)
Anti-WNT10A antibody	Novus Biologicals	NBP1-76916	1:1000 (WB)
HRP anti-goat	Santa Cruz	Sc-2020	1:5000 (WB)
HRP anti-mouse	Cell Signaling Technology	7076S	1:6000 (WB)
HRP anti-rabbit	Cell Signaling Technology	7074S	1:6000 (WB)
Rabbit IgG	Diagenode	C15410206	2 µg (IP)
Anti-EGFP antibody	Abcam	ab13970	1:1000 (IF)
Anti-IL-6 antibody	Abcam	ab6672	1:50 (IF)
Anti-IL-6 antibody	Biolegend	504503	1: 20 (FC)
Anti-E-Cadherin antibody	BD Bioscience	610181	1:100 (IF)
Goat anti-rabbit Alexa Fluor 488	ThermoFisher Scientific	A-11008	1:500 (IF)
Goat anti-mouse Alexa Fluor 568	ThermoFisher Scientific	A-21124	1:500 (IF)
Donkey anti-goat Alexa Fluor 568	ThermoFisher Scientific	A-11057	1:500 (IF)

Supplemental Table 3

Chang-Seng-Yue: Basal-A	Chang-Seng-Yue: Basal-B
PGF	CFAP45
ADM	RHEBL1
PANX2	ZMYND10
PIK3R3	RIBC2
HAS3	SLC4A11
GATSL3	GINS2
ADRB2	BDNF
FAM127C	PLA2G16
LY6K	PPP1R1C
PLEKHF1	SKAP1
ZNF385A	ANXA9
ANKRD33B	C11orf63
EYA2	TNNI3
FAM212B	MLF1
ADORA2B	ANKRD1
CHST7	LRRC73
ADA	RAMP1
SNCG	PBK
TFAP2C	KRT80
S100A2	C9orf116
FJX1	CFAP57
CLEC2B	CHRNA5
HSPB1	PIFO
TNNT1	TRIM59
SLC7A5	CRIP2
ZIC2	FOSL1
FAM225A	TTLL9
BASP1	C2orf81
HSPB8	TCTEX1D2
STC2	MRPS17
GALNT18	VSIG10L
LAG3	THAP10
FBXO27	CCDC74B
P2RY2	DKK1
CTSV	TRIM36
IL31RA	BIRC3
KRT15	VSTM2L
ZP3	FSIP1
RHOV	KCNH3
PACSIN3	LRRC46
SCML2	MNS1
KLC3	NRP2
ULBP2	BATF2
S100A3	FBXO24
CBX2	KIAA1549L

SLCO1B3	CLDN9
ANXA1	AP1S2
TUBB6	NXNL2
ALOXE3	E2F7
GPR19	SPTBN5
RHCG	C7orf57
FOXD1	SPAG8
FAM46B	MTAP
CRABP2	RARRES3
PLTP	CDKL2
NXN	MALL
TMEM40	DNAAF3
TCF7L1	CFP
NUPR1	FRMD5
GGH	SH3TC2
MME	RASAL2-AS1
SLC2A9	TGM2
LRFN1	L1CAM
EPHB2	TMEM169
SCPEP1	F3
TRIM6	PCDH7
ST20	OXTR
PARVB	MYBL1
NRG1	C15orf48
CERS4	IFITM1
NKX3-1	ELFN2
A4GALT	EFHB
TP73	WNT10A
SIX1	C15orf62
NDRG1	C11orf70
MCTP1	RAB36
MT1X	HERC5
C9orf84	DNAH3
CAV1	IFNE
CAMK1D	OAS2
RYR1	C15orf52
ABCA4	MX1
MAPK11	CCDC114
ADARB1	CFAP157
AQP3	AMIGO2
FAAP24	IFIT1
NDUFA4L2	DCBLD2
DYNC1I1	PMAIP1
DMKN	
MT2A	
SCNN1B	
C17orf53	
PHGDH	

ALDH1A3
NBPF26
C12orf60
PPP2R2C
ETNK2
TBX1
PLAU
FBXO48
CLEC2D
WNT5B
HS6ST1
RFESD
ANGPTL4
SEMA3F
SYT7
STOM
SCHIP1
KCNIP3
FANCB
EREG
FSCN1
ATP8B3
GCLM
HMGA2
HAP1
ESPN
TMEM132A
HES2
GPNMB
FIGN
FST
SYNM
GS1-259H13.2
ITGA2B
GJB2
P2RY6
SLC22A4
PYGL
GJB6
LYNX1
XDH
AKR1C1
PFN2
NETO2
GNAI1
IL20RB
KCNS3
EDARADD

CFAP58
ARSJ
TOX2
TLR6
FGFR3
CD109
STARD5
RP11-382A20.3
TIAM1
CPA4
HACD1
RNF217
SHROOM2
LAMP3
GPR155
TMEM156
ADSSL1
SLC39A8
B3GNT4
C11orf45
KCNG1
TMEM45A
KRT13
SRPX2
CSNK2A3
VWDE
FABP5
EME1
KRT81
SRGAP3
EVA1A
FAM126A
FSTL4
CNTNAP3
BTBD11
C18orf54
IL27RA
NGFR
MCC
PRSS27
LPAR3
SEMA3A
FAM169A
DNAH17
LDLRAD3
CHST15
HSF2BP
PTX3

CYP27C1
PALMD
RDX
COL7A1
DSC3
CDH26
TGM1
WNT7B
PTGES
ARL4D
KRT6A
MAPK12
CORO6
RYR3
KRT5
SLITRK6
FAM83A
IRX3
PTPN13
PROM2
TGFBI
CACHD1
PTHLH
LCP1
PDZD2
TP63
TENM2
COL4A5
FAT2
EMP1
PSTPIP2
MTSS1
KLHL13
FGFBP1
APOBEC3G
DNER
NT5M
SCNN1G
FLG
WNT9A
ZIC5
CDH3
ICAM5
DFNA5
SLC16A14
TNC
SIX4
ST6GALNAC2

ADIRF-AS1
CYP26B1
FHOD3
SNAI2
SLC37A2
THBD
KDM4D
